# Author Correction: Learning perturbation-inducible cell states from observability analysis of transcriptome dynamics

**DOI:** 10.1038/s41467-024-46433-2

**Published:** 2024-03-06

**Authors:** Aqib Hasnain, Shara Balakrishnan, Dennis M. Joshy, Jen Smith, Steven B. Haase, Enoch Yeung

**Affiliations:** 1 Department of Mechanical Engineering, University of California Santa Barbara, Santa Barbara, CA USA; 2 Department of Electrical and Computer Engineering, University of California Santa Barbara, Santa Barbara, CA USA; 3 California Nanosystems Institute, University of California Santa Barbara, Santa Barbara, CA USA; 4 Department of Biology, Duke University, Durham, NC USA

**Keywords:** Systems analysis, Synthetic biology, Dynamical systems, Time series

Correction to: *Nature Communications* 10.1038/s41467-023-37897-9, published online 31 May 2023

In the Acknowledgement section of the article, the following grant numbers were inadvertently omitted:

- The grant numbers FA8750-17-C-0229, HR001117C0092, HR001117C0094, and DEAC0576RL01830 relating to the Air Force Research Laboratory.
- The grant number 545157 relating to Pacific Northwest National Laboratories.
- The grant number W911NF-20-1-0165 relating to the Army Research Office Young Investigators Program Award.
- The grant numbers W911NF-19-0026, W911NF-19-F-0037, and W911NF-19-D-0001 relating to the Institute of Collaborative Biotechnologies.

The original article has been corrected.

