# Supplementary material for: Learning perturbation-inducible cell states from observability analysis of transcriptome dynamics
Source: Nat Commun. 2023 May 31;14:3148. doi: 10.1038/s41467-023-37897-9 (PMC10229592; doi:10.1038/s41467-023-37897-9)
Supplement: Supplementary file 1 — Supplementary Information [file 41467_2023_37897_MOESM1_ESM.pdf]

Learning perturbation-inducible cell states of novel compounds from observability analysis of transcriptome dynamics

Aqib Hasnain et al.

1 Supplementary Text

1.1 Observability maximization for transcriptome dynamics

Here we derive the solution to the observability maximization problem briefly outlined in the Methods section. Recall that we have a state-space representation of the transcriptome dynamics as

$$\begin{aligned} \mathbf{x}_{t+1} &= \mathbf{K}\mathbf{x}_t \\ \mathbf{y} &= \mathbf{W}\mathbf{x}_t \end{aligned} \tag{1}$$

where $\mathbf{x} \in \mathbb{R}^n$ is the (hidden) cell state, $\mathbf{K}$ is the state transition matrix, $\mathbf{W}$ are the unknown gene sampling weights, and $\mathbf{y} \in \mathbb{R}^p$ are the p measurements. The objective, $\mathcal{J}$, is formulated by the signal energy (or output energy) of the system

$$\mathcal{J} = \sum_{i=1}^{m} \mathbf{y}_i^\top \mathbf{y}_i = \sum_{i=0}^{m} \mathbf{x}_0^\top \mathbf{K}^{i^\top} \mathbf{W}^\top \mathbf{W} \mathbf{K}^i \mathbf{x}_0, \tag{2}$$

and we seek the gene sampling weights $\mathbf{W}$ which maximize the objective

$$\begin{aligned} &\max_{\mathbf{W} \in \mathbb{R}^{p\times n}} \mathcal{J} \\ &\text{subject to } \mathbf{W}\mathbf{W}^\top = I_{p\times p}. \end{aligned} \tag{3}$$

The constraint enforces that the rows of $\mathbf{W}$ are orthogonal to each other and that the length of each row be equal to 1. This further avoids the issue of the objective blowing up to infinity. The solution to the above optimization problem is obtained by forming the Lagrangian dual problem and finding the maxima of the the dual objective in terms of the dual variable (a $p \times p$ matrix), $\mathbf{D}$, i.e.

$$\begin{aligned} &\max_{\mathbf{W} \in \mathbb{R}^{p\times n}} \mathcal{J} + \mathcal{L} \\ &\text{where } \mathcal{L} = -\text{tr}\Big((\mathbf{W}\mathbf{W}^\top - I_{p\times p})\mathbf{D}\Big) \end{aligned} \tag{4}$$

and tr() denotes the trace operator. Differentiating the dual objective with respect to $\mathbf{W}^\top$ and equating to 0, we have

$$\begin{aligned} \frac{\partial(\mathcal{J}+\mathcal{L})}{\partial \mathbf{W}^\top} &= \frac{\partial}{\partial \mathbf{W}^\top}\left(\sum_{i=0}^{m} \mathbf{x}_0^\top \mathbf{K}^{i^\top} \mathbf{W}^\top \mathbf{W} \mathbf{K}^i \mathbf{x}_0 - \text{tr}\Big((\mathbf{W}\mathbf{W}^\top - I_{p\times p})\mathbf{D}\Big)\right) \\ &= \frac{\partial}{\partial \mathbf{W}^\top}\left(\sum_{i=0}^{m} \text{tr}(\mathbf{x}_0^\top \mathbf{K}^{i^\top} \mathbf{W}^\top \mathbf{W} \mathbf{K}^i \mathbf{x}_0) - \text{tr}\Big((\mathbf{W}\mathbf{W}^\top - I_{p\times p})\mathbf{D}\Big)\right) \\ &= \frac{\partial}{\partial \mathbf{W}^\top}\left(\sum_{i=0}^{m} \text{tr}(\mathbf{W} \mathbf{K}^i \mathbf{x}_0 \mathbf{x}_0^\top \mathbf{K}^{i^\top} \mathbf{W}^\top) - \text{tr}\Big((\mathbf{W}\mathbf{W}^\top - I_{p\times p})\mathbf{D}\Big)\right) \\ &= \frac{\partial}{\partial \mathbf{W}^\top}\left(\sum_{i=0}^{m} \text{tr}(\mathbf{W}\mathbf{G}^{(i)}\mathbf{W}^\top) - \text{tr}\Big((\mathbf{W}\mathbf{W}^\top - I_{p\times p})\mathbf{D}\Big)\right) \\ &= \frac{\partial}{\partial \mathbf{W}^\top}\left(\text{tr}(\mathbf{W}\sum_{i=0}^{m}\mathbf{G}^{(i)}\mathbf{W}^\top) - \text{tr}\Big((\mathbf{W}\mathbf{W}^\top - I_{p\times p})\mathbf{D}\Big)\right) \\ &= \frac{\partial}{\partial \mathbf{W}^\top}\left(\text{tr}(\mathbf{W}\mathbf{G}\mathbf{W}^\top) - \text{tr}\Big((\mathbf{W}\mathbf{W}^\top - I_{p\times p})\mathbf{D}\Big)\right) \\ &= 2\mathbf{G}\mathbf{W}^\top - 2\mathbf{W}^\top\mathbf{D} = 0 \end{aligned} \tag{5}$$

where the second equality comes from the fact that $\mathcal{J}$ is a sum of m scalars and so applying the trace operator has no effect on the sum, the third equality uses the cyclic property of the trace of products, and the fifth equality uses the fact that $\text{tr}(\mathbf{A}) + \text{tr}(\mathbf{B}) = \text{tr}(\mathbf{A} + \mathbf{B})$. Finally, the Gram matrix, $\mathbf{G}$, is defined to be $\mathbf{G} = \sum_i \mathbf{G}^{(i)} = \sum_i \mathbf{K}^i \mathbf{x}_0 \mathbf{x}_0^\top \mathbf{K}^{i^\top}$, a sum of quadratic forms, which is itself a quadratic form and therefore a symmetric matrix with non-negative, real-valued eigenvalues. From the final equality in Eq. (5) we have

$$\mathbf{G}\mathbf{W}^\top = \mathbf{W}^\top \mathbf{D} \tag{6}$$

which says columns of the eigenvectors of $\mathbf{G}$ are the rows of gene sampling weights $\mathbf{W}$. Moreover, the eigenvector of $\mathbf{G}$ corresponding to the eigenvalue with largest magnitude in $\mathbf{D}$ is the maximizer when $p = 1$.

1.2 Maximizing observability - the integer program

Given the same state-space representation as before, in this section we will develop the mixed-integer program version of the sensor placement framework and highlight the specific relaxations that were made to be able to formulate an analytically tractable optimization problem. We again start by seeking to maximize the signal energy $\mathcal{J}$, however we now add several additional constraints

$$\begin{aligned} &\max_{\mathbf{W} \in \{0,1\}^{p \times n}} \mathcal{J} \\ &\text{subject to } \sum_{i=1}^{n} \mathbf{W}_{ki} = 1, \ k = 1, \dots, p. \end{aligned} \tag{7}$$

The additional constraints we have added on the optimization problem are i) $\mathbf{W}$ is now a binary matrix, representing whether a gene should be sampled or not, ii) each measurement y_i, $i = 1, \dots, p$ can only be a measurement of a single gene. This problem is clearly an integer programming and in general is difficult to solve for the optimal matrix $\mathbf{W}$. In the section above, we have relaxed both of these constraints, allowing $\mathbf{W}$ to take values in the real numbers and make no restriction on the amount of genes that each measurement can sample from. These relaxations turn the problem from computationally intractable to analytically tractable and the computation time depends largely on an eigendecomposition of $r \times r$ matrices (if using DMD approximations of the network and Gram matrices) or of $n \times n$ matrices with n on the order of 1000 for genetic networks.

1.3 A brief exposition of observability maximization on simulated linear systems

To more clearly emphasize the effect of network topology on the observability maximization problem, in this section we explore the structure of the learned sampling weights as the connectivity of the state-transition matrix, $\mathbf{K}$, is varied for simulated linear systems. We generate fully-connected and sparse block-diagonal matrices to act as the state-transition matrices and for each, we further explore how the number of time points used in the optimization problem (2) affects the learned weights, $\mathbf{w}$.

We simulated 5 distinct network topologies (state-transition matrices) for a simulated 50-dimensional linear system, i) fully-connected, ii) 5 blocks (each fully-connected), iii) 10 blocks, iv) 25 blocks, v) 50 blocks (fully decoupled). For each topology, we obtained the sampling weights that maximize observability for number of time points, $T = 2$ and $T = 5$. Supplementary Figure 1a shows the sampling weights corresponding to each state variable for each topology and time point. For fully-connected topologies, we find that sampling any state variable (gene) for any number of time points leads to similar observability of the entire system, i.e. no one variable contains more information for state reconstruction. As the system transitions from fully-connected to decoupled, key genes are highlighted by higher magnitude weights. The second row in Supplementary Figure 1a corresponds to the learned weights and the state-transition matrices while the third row corresponds to the sorted magnitude of the weights. We can see that as T is increased, the block-diagonal systems' weights transition to more exponential-like curves, resulting in a clear threshold between variables which contribute to observability and variables which do not.

Next, we simulated the same 5 systems as above, however we now set the final (20,20) submatrix to zero. This results in a completely decoupled set of state variables for the last four systems, while for the first system it results in a lack of self edges from the submatrix state variables to themselves. For each topology, we obtained the sampling weights that maximize observability for number of time points, $T = 2$ and $T = 5$. Supplementary Figure 1b shows the sampling weights corresponding to each state variable for each topology and time point. For the first system, we see that lack of self-edges for the lower submatrix results in lower magnitude weights for the corresponding state variables. This discrepancy diminishes as we move from $T = 2$ to $T = 5$ due to information transfer among the genes. For the remaining four systems, we see the same behavior as before, however now the weights corresponding to the state variables with zero submatrices are exactly or nearly zero. As T is increased, we see that the zero submatrix weights converge to zero – information does not transfer

to these genes. Initially, for small T, the weights are not exactly zero due to nonzero initial conditions. As the initial condition is propagated further in time, its contribution is diminished and the weights converge to zero.

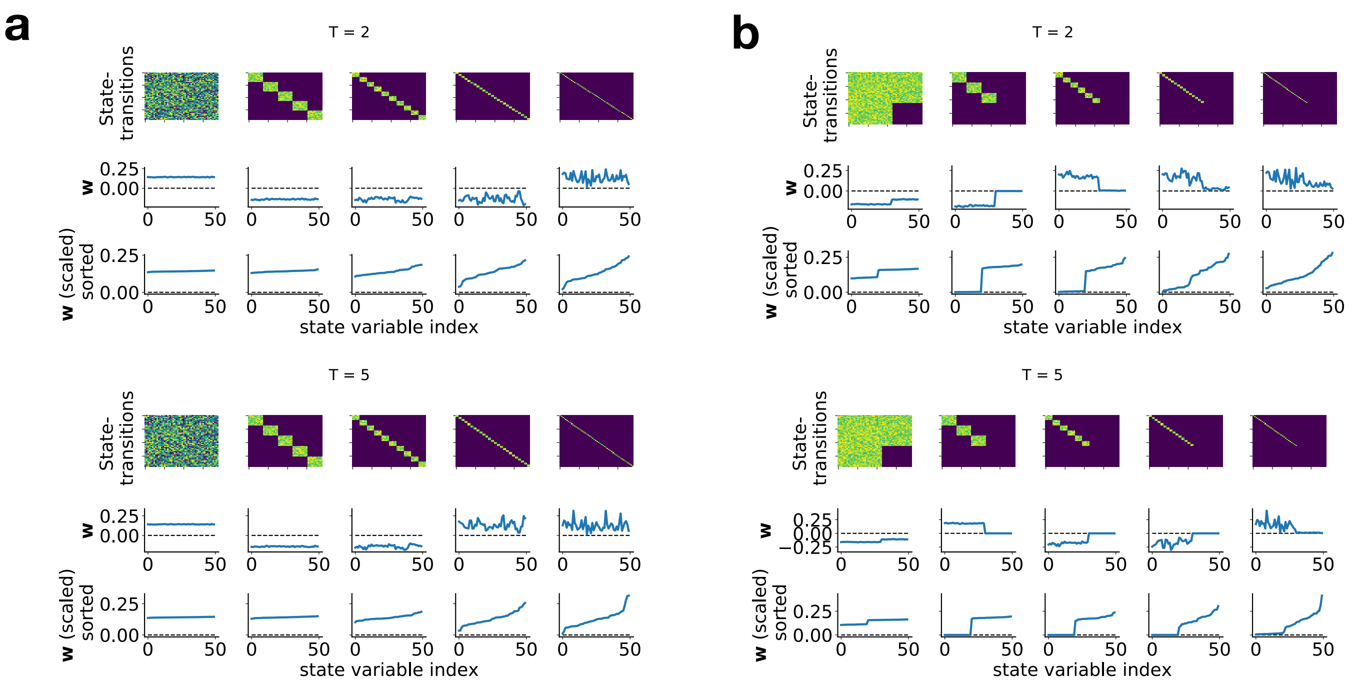

Figure 1: **(a)** From right to left, five systems are simulated and gene sampling weights are obtained and plotted. The system state-transition matrices are shown in the first row, the sampling weights are shown in the second row, and the sorted magnitude of the sampling weights are shown in the third row. **(b)** Same as (a), however now the final (20,20) submatrix of each system is set to zero.

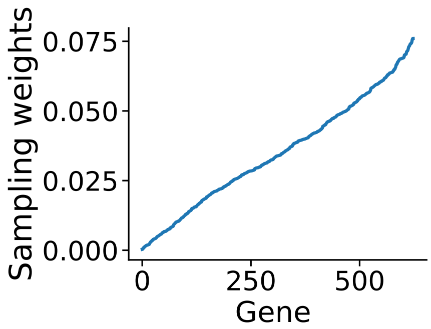

Figure 2: The gene sampling weights that maximize observability for the transcriptomic response of SBW25 to malathion.

For the transcriptomic response of SBW25 to malathion (Supplementary Figure 2), the gene sampling weights are in the regime of nonzero initial conditions for a majority of the genes, nearly fully connected, and relatively large T ($T = 8$). The contribution of each of the above points result in a continuum of sampling weights in which no group of genes is ranked highly compared to the rest of the genes. Notably, even though the learned state-transition matrix is fully connected, we are able to recover a range of sampling weights for the 624 genes. Conversely, for simulated fully-connected networks, we are unable to identify important differences from the sampling weights (the weights are uniformly spread). Consequently, we can conlude that our novel observability analysis is able to capture observable genes from transcriptomic datasets.

1.4 Fold change dynamics of two linear systems

The gene expression dynamics of each experimental condition (negative control and malathion) are well approximated by a linear state-space representation. Specifically, using 10 DMD, we obtained an n-step accuracy of $R^2 = 0.99$ for both conditions. Given this is the case, in this section we want to show when the fold change dynamics of two linear systems

can be represented by a linear system. We then define the dynamics as

$$\begin{aligned} \frac{dx_{\text{on}}}{dt} &= ax_{\text{on}} + bu \\ \frac{dx_{\text{off}}}{dt} &= ax_{\text{off}} \end{aligned} \tag{8}$$

where here x_{on} and x_{off} are scalar variables for ease of analysis. The variables represent the dynamics in the case where the input is present (*on*) and when the input is absent (*off*), respectively. The input u represents the scalar input of a small molecule, e.g. malathion, that drives the expression of genes in the *on* condition through a step input, i.e. $u(t) = 1$ for all $t > 0$. The solution of the linear ordinary differential equations above are given by

$$\begin{aligned} x_{\text{on}}(t) &= e^{at}x_0 + \int_0^t e^{a(t-\tau)} bu(\tau) d\tau \\ x_{\text{off}}(t) &= e^{at}x_0 \end{aligned} \tag{9}$$

where $x(0) = x_0$ for both x_{on} and x_{off}. We want to show that the fold change response is given by the solution of a linear dynamical system. Taking the fold change of x_{on} to x_{off} we have

$$\begin{aligned} x_{\text{fc}}(t) = \frac{x_{\text{on}}}{x_{\text{off}}}(t) &= 1 + \int_0^t e^{-a\tau} \frac{b}{x_0} d\tau \\ &= 1 + \frac{b}{ax_0} - \frac{b}{ax_0} e^{at} \\ &= 1 + \alpha - \alpha e^{at}. \end{aligned} \tag{10}$$

To show that there exists a linear ordinary differential equation (ODE) that gives rise to the above solution $x_{\text{fc}}(t)$, we apply the steps to solve linear ODEs using integrating factors but in reverse order. We know in advance that the integrating factor should take the form e^{at} and we start by dividing both sides of (10) by this integrating factor

$$e^{-at}x_{\text{fc}} = e^{-at}(1+\alpha) - \alpha. \tag{11}$$

We next differentiate both sides and integrate both sides with respect to t

$$\int_0^t \frac{d}{dt}\Big(e^{-at}x_{\text{fc}}\Big) dt = \int_0^t ae^{-at} dt - \int_0^t \alpha a e^{-at} dt, \tag{12}$$

then once again differentiating both sides gives

$$\frac{d}{dt}\Big(e^{-at}x_{\text{fc}}\Big) = ae^{-at} - \alpha a e^{-at}. \tag{13}$$

Applying the product rule to the left hand side, we have

$$\begin{aligned} e^{-at}\frac{dx_{\text{fc}}}{dt} - ae^{-at}x_{\text{fc}} &= ae^{-at} - \alpha a e^{-at} \\ &= e^{-at}(a - \alpha a). \end{aligned} \tag{14}$$

Finally, multiplying through by the integrating factor, e^{at}, and solving for $\frac{dx_{\text{fc}}}{dt}$, we obtain

$$\frac{dx_{\text{fc}}}{dt} = ax_{\text{fc}} + a - \alpha a \tag{15}$$

which is a linear first order ODE, i.e. a linear dynamical system with a step input and $\alpha = \frac{b}{ax_0}$. The importance of this result is to to be able to say that if the dynamics of the transcriptome in each experimental condition are well represented by a linear system, than the fold change dynamics, under the stated assumptions, can also be well represented by a linear system.

We briefly remark on the extension to the multivariate case. Under the assumption that the system dynamics, A, is diagonalizable, the above analysis holds. One such transformation which diagonalizes the the dynamics is given by the set of eigenvectors of A. Formally, if we now have system dynamics with state, $\mathbf{x} \in \mathbb{R}^n$, such that

$$\begin{aligned} \frac{d\mathbf{x}_{\text{on}}}{dt} &= A\mathbf{x}_{\text{on}} + Bu \\ \frac{d\mathbf{x}_{\text{off}}}{dt} &= A\mathbf{x}_{\text{off}}, \end{aligned} \tag{16}$$

applying the transformation $\tilde{\mathbf{x}} = T^{-1}\mathbf{x}$, where $T \in \mathbb{R}^{n\times n}$ is the matrix of eigenvectors of A, results in the transformed systems

$$\begin{aligned}\frac{d\tilde{\mathbf{x}}_{\text{on}}}{dt} &= D\tilde{\mathbf{x}}_{\text{on}} + \tilde{B}u \\ \frac{d\tilde{\mathbf{x}}_{\text{off}}}{dt} &= D\tilde{\mathbf{x}}_{\text{off}},\end{aligned} \tag{17}$$

where $\tilde{B} = T^{-1}B$. To solve for the fold change dynamics in the multivariate case, we cast the state coordinates into a diagonal matrix, i.e. $\text{diag}(\tilde{\mathbf{x}})$, and compute $\text{diag}(\tilde{\mathbf{x}}_{\text{on}})(\text{diag}(\tilde{\mathbf{x}}_{\text{off}}))^{-1}$. Since the solution in each coordinate is uncoupled from other coordinates, we then have n solutions, each as in Eq. (10).

The case where the above derivation does not hold when the eigenvalues of A have zero real part, i.e. they are exactly zero or have purely sinusoidal response (corresponding to periodic orbits). In this case, the fold change in the coordinate corresponding to zero eigenvalues will approach infinity or it will not be possible to represent the fold change dynamics as a sum of weighted exponentials, e.g. $\tan(x)$. However, such a case would be improbable in a data-driven application for gene regulatory networks. Moreover, any eigenvalue with magnitude zero corresponds to fast dynamics that decay quickly, not significantly contributing to the overall dynamics. There is a time-scale separation between low magnitude and high magnitude eigenvalues, and low magnitude eigenvalues may be removed with little to no effect on the dynamics.

2 Supplementary Figures

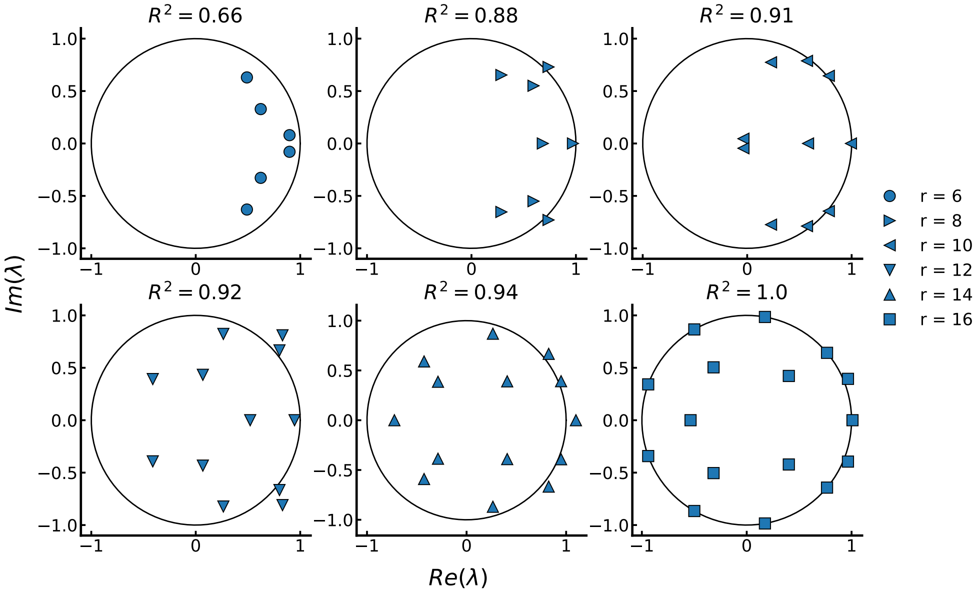

Figure 3: The eigenvalues of the DMD operator plotted in the complex plane for varying number of modes.

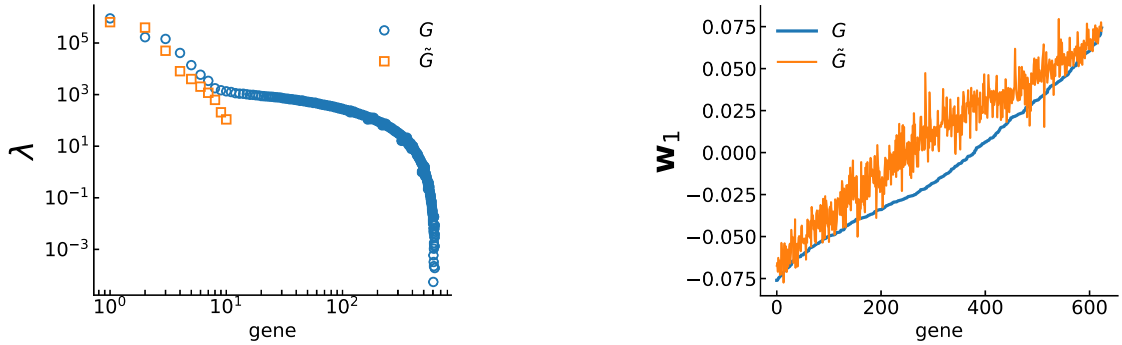

Figure 4: (Left) Approximation of the eigenvalues of the Gram matrix by the reduced order model given by DMD. The full Gram matrix eigenvalues are given in blue circles and the reduced Gram matrix eigenvalues are given in orange squares. (Right) Approximation of the leading eigenvector of the Gram matrix by the reduced order model given by DMD. This eigenvector corresponds to the gene sampling weights in the main text.

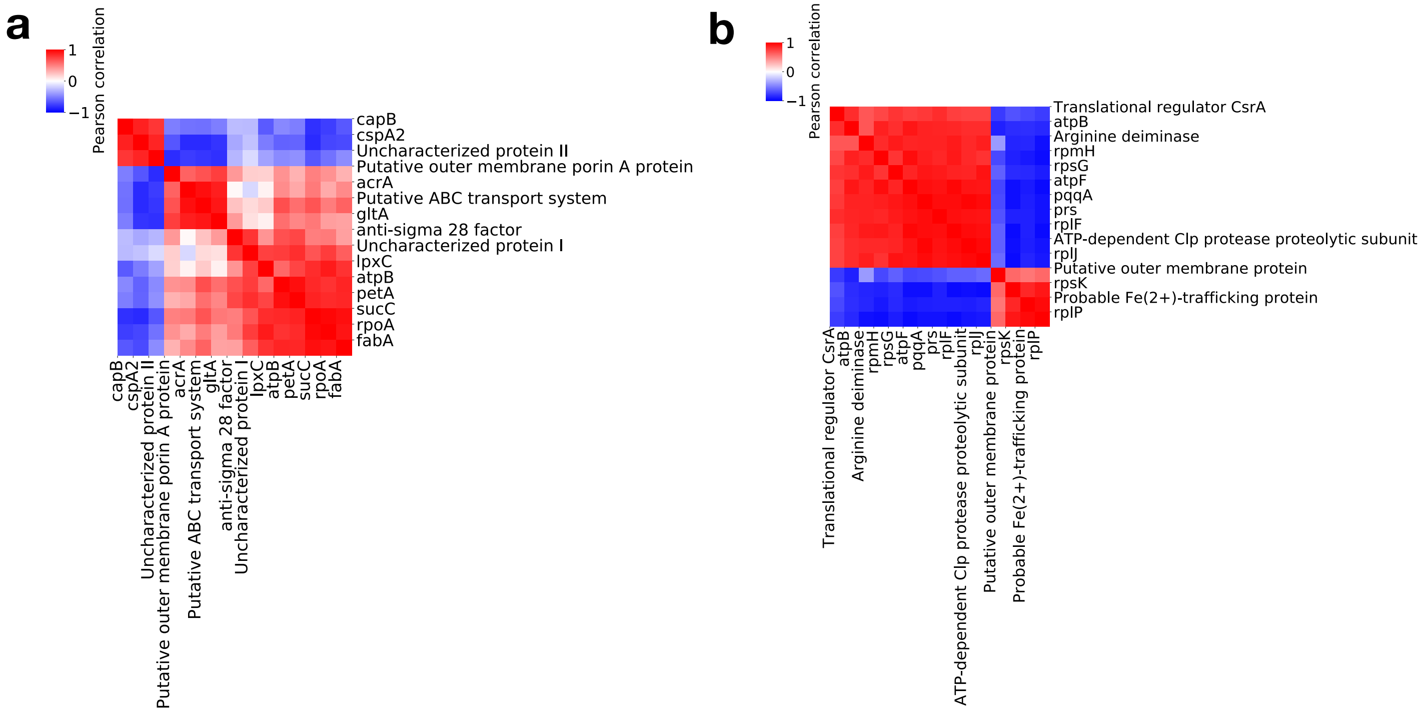

c

locus_tag	gene	protein_name	molecular function	biological process
PFLU_6136	rpmH	50S ribosomal protein L34	structural constituent of ribosome	translation
PFLU_5860	N/A	Putative outer membrane protein	N/A	N/A
PFLU_3929	N/A	ATP-dependent Clp protease proteolytic subunit	ATP-dependent peptidase activity; serine-type endopeptidase activity	proteolysis
PFLU_4746	csrA	Translational regulator CsrA	mRNA 5'-UTR binding	regulation of translational initation; regulation of carbohydrate metabolic process
PFLU_6124	atpB	ATP synthase subunit a	proton-transporting ATP synthase activity, rotational mechanism	N/A
PFLU_5504	rpsK	30S ribosomal protein S11	structural constituent of ribosome	translation
PFLU_5512	rplF	50S ribosomal protein L6	structural constituent of ribosome	translation
PFLU_5536	rplJ	50S ribosomal protein L10	structural constituent of ribosome	translation
PFLU_6122	atpF	ATP synthase subunit b	hydrolase activity; proton-transporting ATP synthase activity, rotational mechanism	N/A
PFLU_5598	pqqA	Coenzyme PQQ synthesis protein A	N/A	pyrroloquinoline quinone biosynthetic process
PFLU_0732	prs	Ribose-phosphate pyrophosphokinase	ATP binding; kinase activity; magnesium ion binding; ribose phosphate diphosphokinase activiity	5-phosphoribose 1-diphosphate biosynthetic process; nucleotide biosynthetic process; phosphorylation; ribonucleoside monophosphate biosynthetic process
PFLU_0322	N/A	Probable Fe(2+)-trafficking protein	N/A	N/A
PFLU_5531	rpsG	30S ribosomal protein S7	rRNA binding; structural constituent of ribosome; tRNA binding	translation
PFLU_4891	N/A	Arginine deiminase	arginine deiminase activity	arginine catabolic process to ornithine
PFLU_5520	rplP	50S ribosomal protein L16	rRNA binding; structural constituent of ribosome; tRNA binding	translation

Figure 5: **(a)** Pearson correlations between the RNA-seq temporal profiles of the 15 genes in the malathion reporter library. **(b)** Pearson correlations between the RNA-seq temporal profiles of the 15 genes which have the highest observability ranking (sampling weights). **(c)** Metadata associated with the genes in **(b)**.

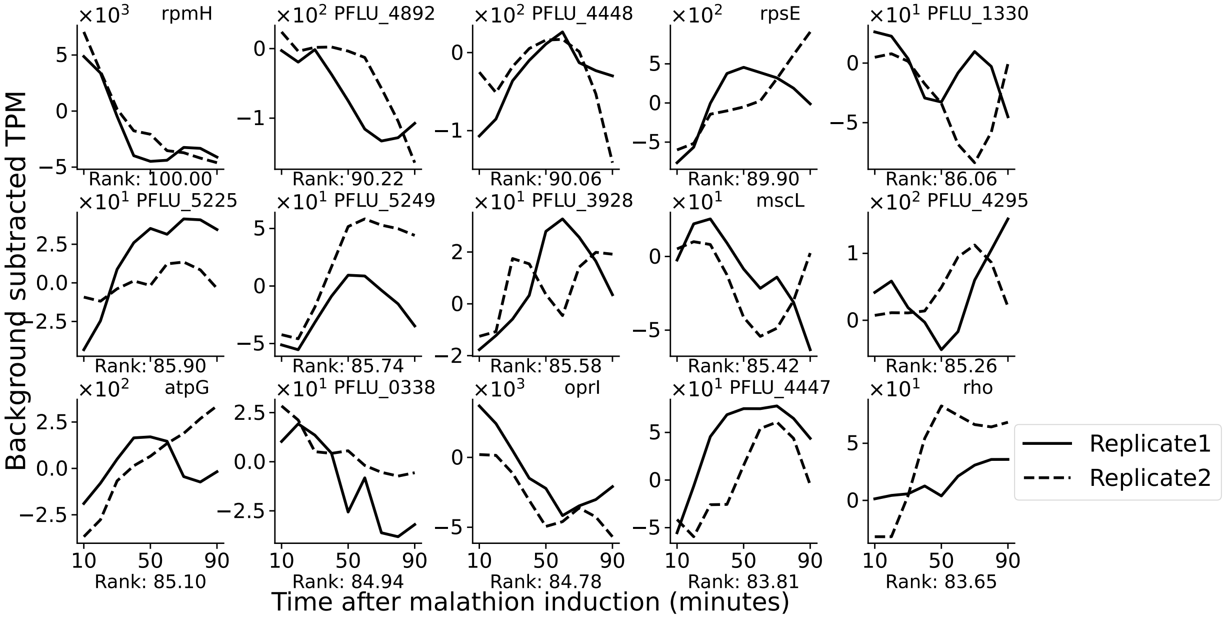

Figure 6: Background (control) subtracted TPM for the 15 genes selected via the correlation selection strategy where the top ranked gene is first selected and any subsequent gene is selected if is lowly correlated with previously selected genes. The correlation threshold used here is 0.5. The x-label of each axis represents the percentage rank out of 624 genes that each gene is assigned by observability maximization.

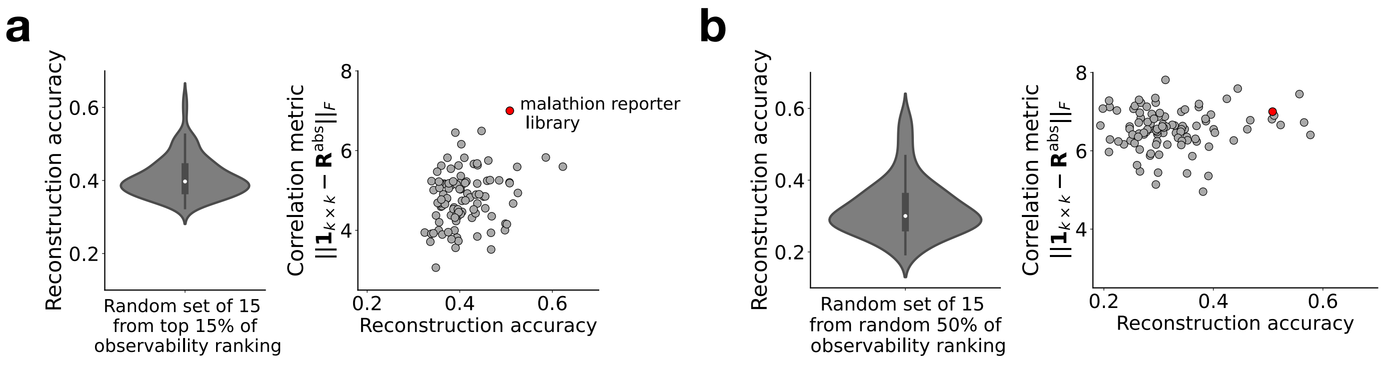

Figure 7: **(a)** (Left) The reconstruction accuracy distribution over random sets of 15 genes selected from the top 15% of observability-ranked genes is visualized with a boxplot. (Right) For each random set of 15 genes, the correlation metric is plotted against the reconstruction accuracy. **(b)** (Left) The reconstruction accuracy distribution over random sets of 15 genes selected from random sets of 312 genes (no matter their rank) is visualized with a boxplot. (Right) For each random set of 15 genes, the correlation metric is plotted against the reconstruction accuracy. The red marker in (a) and (b) marks the reconstruction accuracy and correlation metric for the gene set used to construct the malathion reporter library.

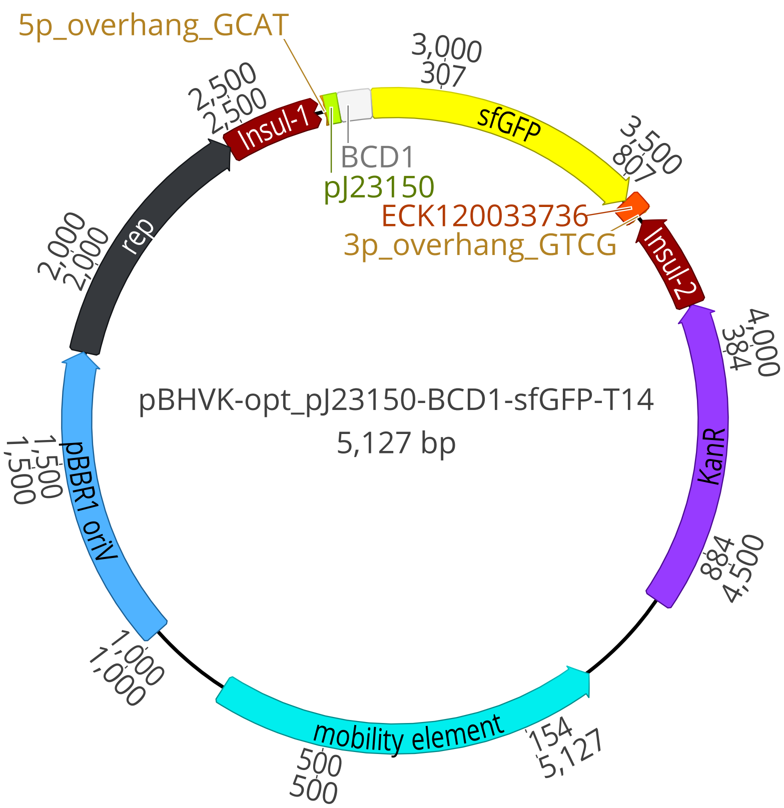

Figure 8: The full plasmid map of pBHVK with the reporter cassette. The two BsaI cut sites on either side of the promoter (5p and 3p overhangs), pJ23150, are used in Golden Gate Assembly to replace the promoter sequence with a promoter used for malathion sensing. A bicistronic design is used for the ribosome binding site, BCD1. A terminator from the set of Voigt lab terminators is used, ECK120033736. For fluorescent reporting, super folder GFP (sfGFP) is used. See Table 3 for sequences of the overhangs, terminator, ribosome binding site, and sfGFP. See Table 2 for sequences of the promoters used in the reporter library.

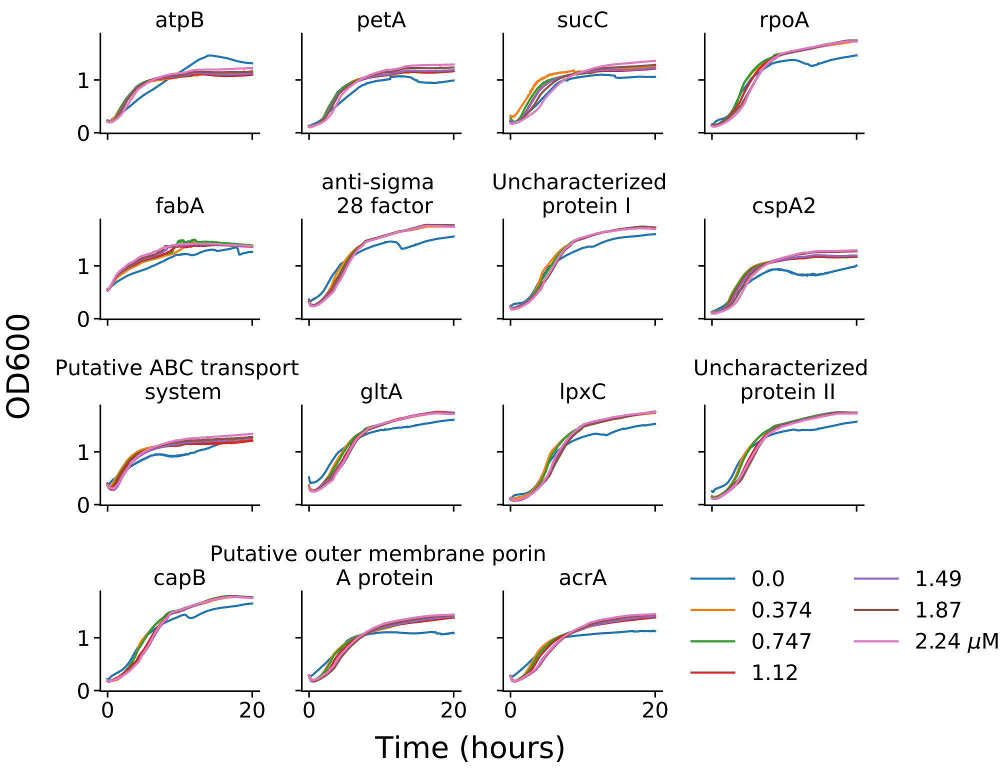

Figure 9: Growth curves of each malathion reporter subject to malathion induction by means of Spectracide.

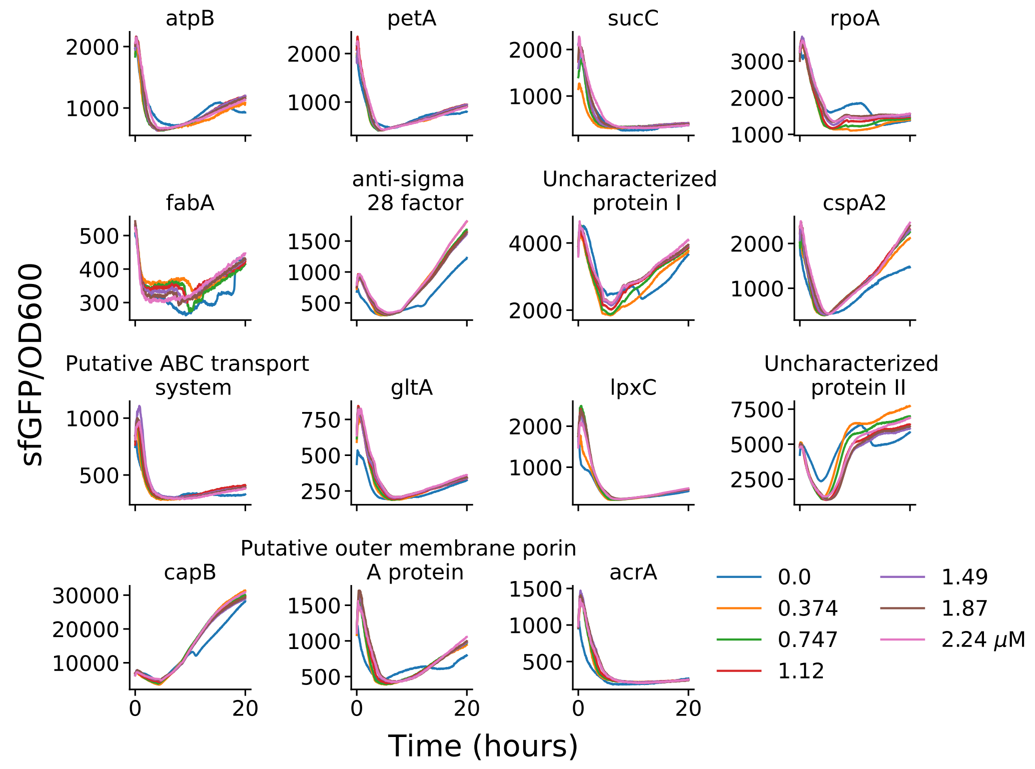

Figure 10: OD normalized sfGFP fluorescence (arbitrary units) of each malathion reporter subject to malathion induction by means of Spectracide.

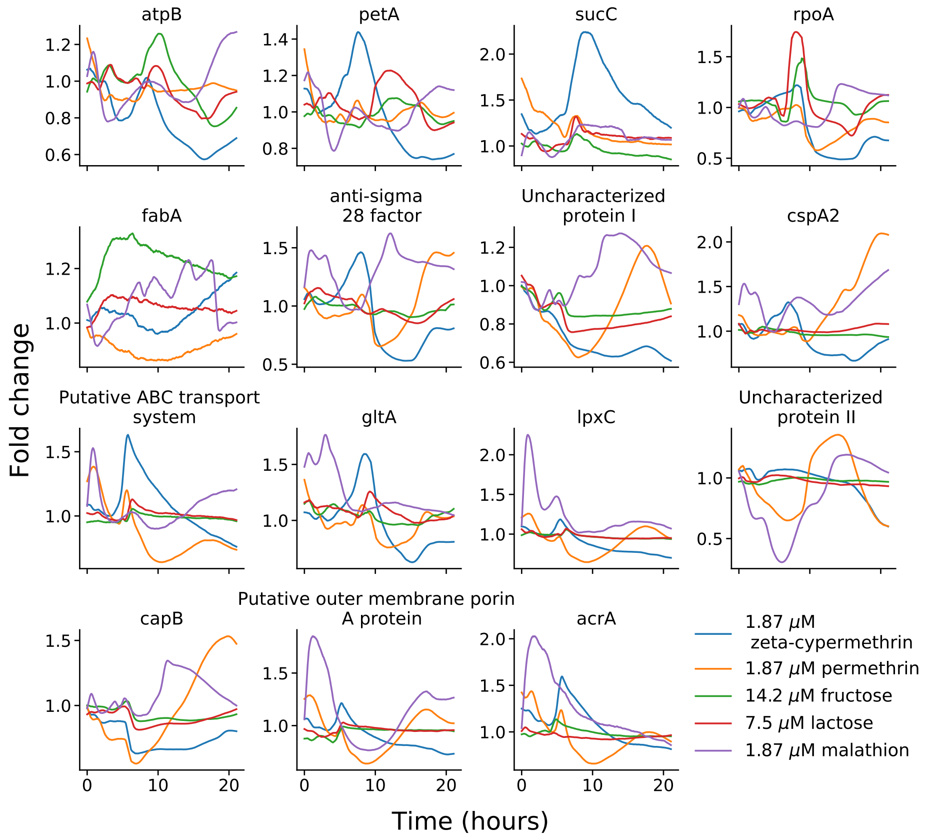

Figure 11: The fold change response of the reporters subjected to five distinct compounds. The fold change is taken as $(\text{sfGFP/OD})_{\text{compound}}/(\text{sfGFP/OD})_{\text{control}}$.

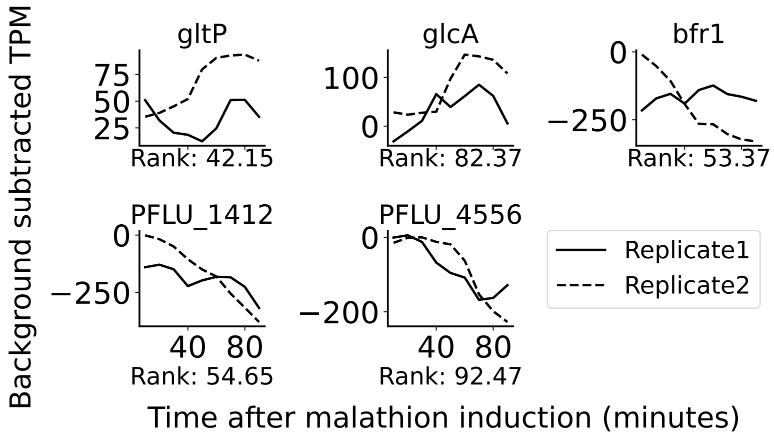

Figure 12: The five differentially expressed genes called by DESeq2 after multiple-testing correction with the Benjamini-Hochberg procedure. The x-label of each axis represents the percentage rank out of 624 genes that each gene is assigned by observability maximization.

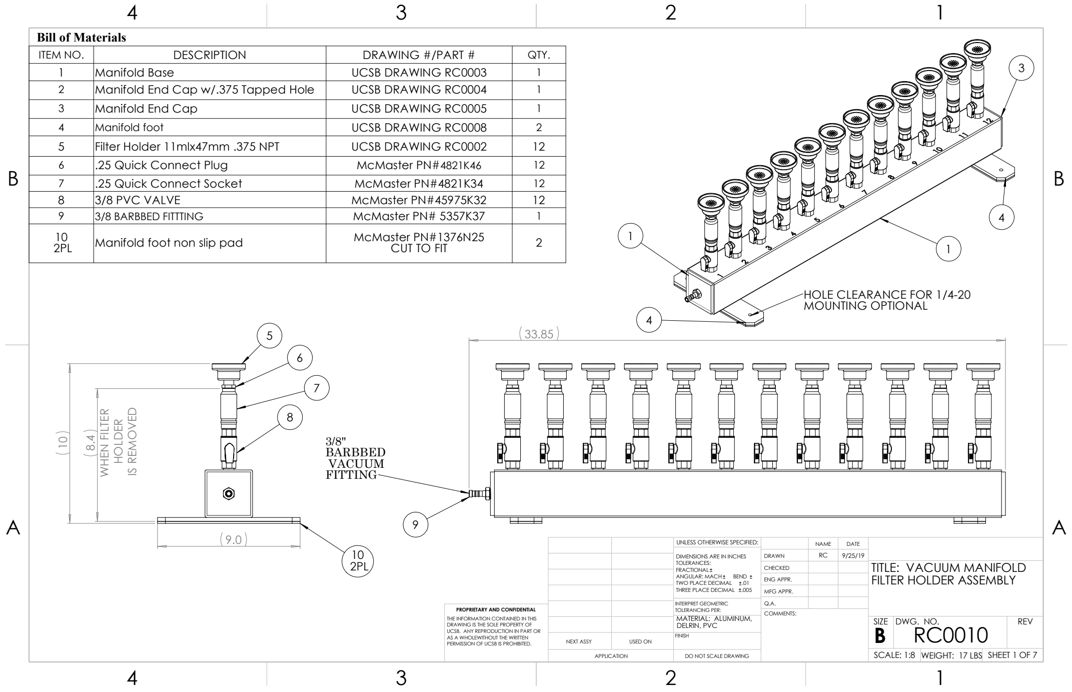

Figure 13: Vacuum manifold design for rapid sampling of mRNA dynamics.

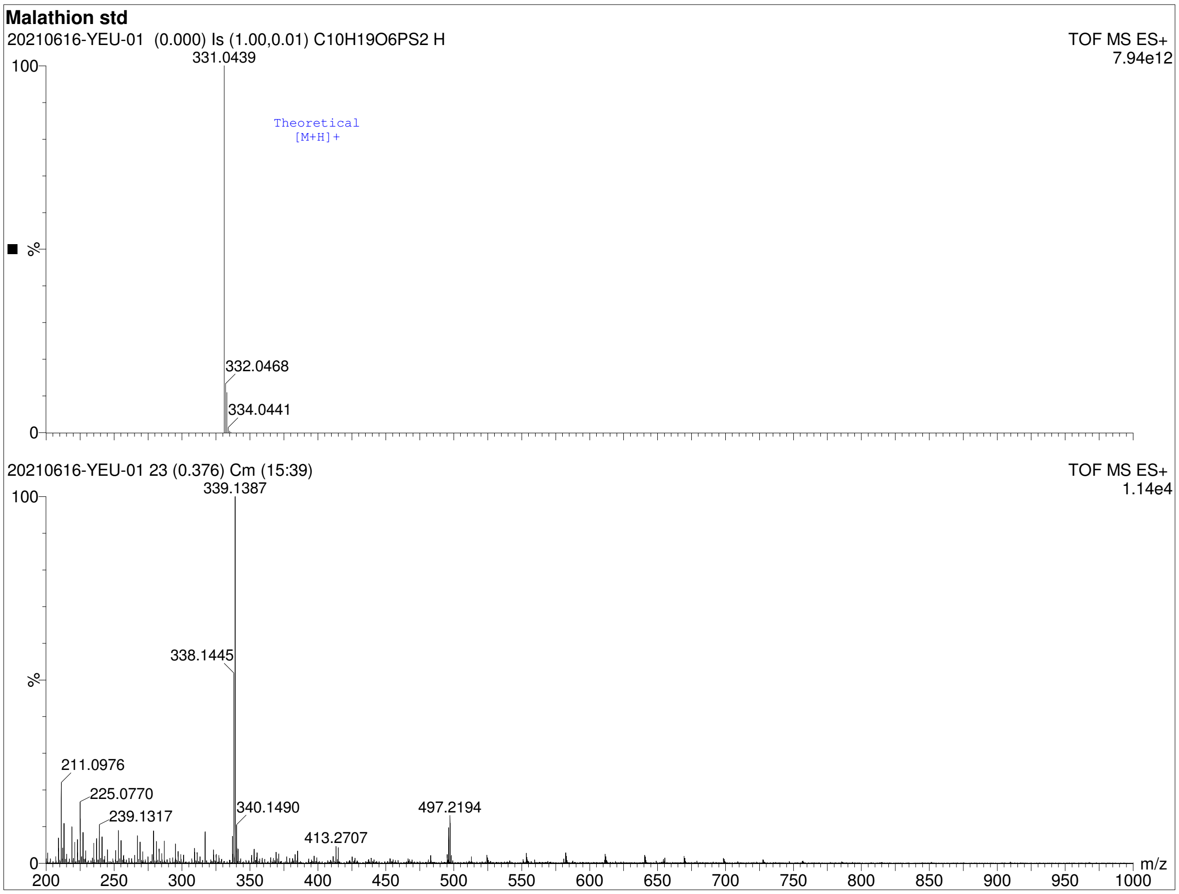

Figure 14: Mass spectrum of malathion (Millipore Sigma Catalog no. 36143) given by time-of-flight mass spectrometry. The theoretical mass spectrum is shown in the upper spectrum and the measured mass spectrum is shown in the lower spectrum.

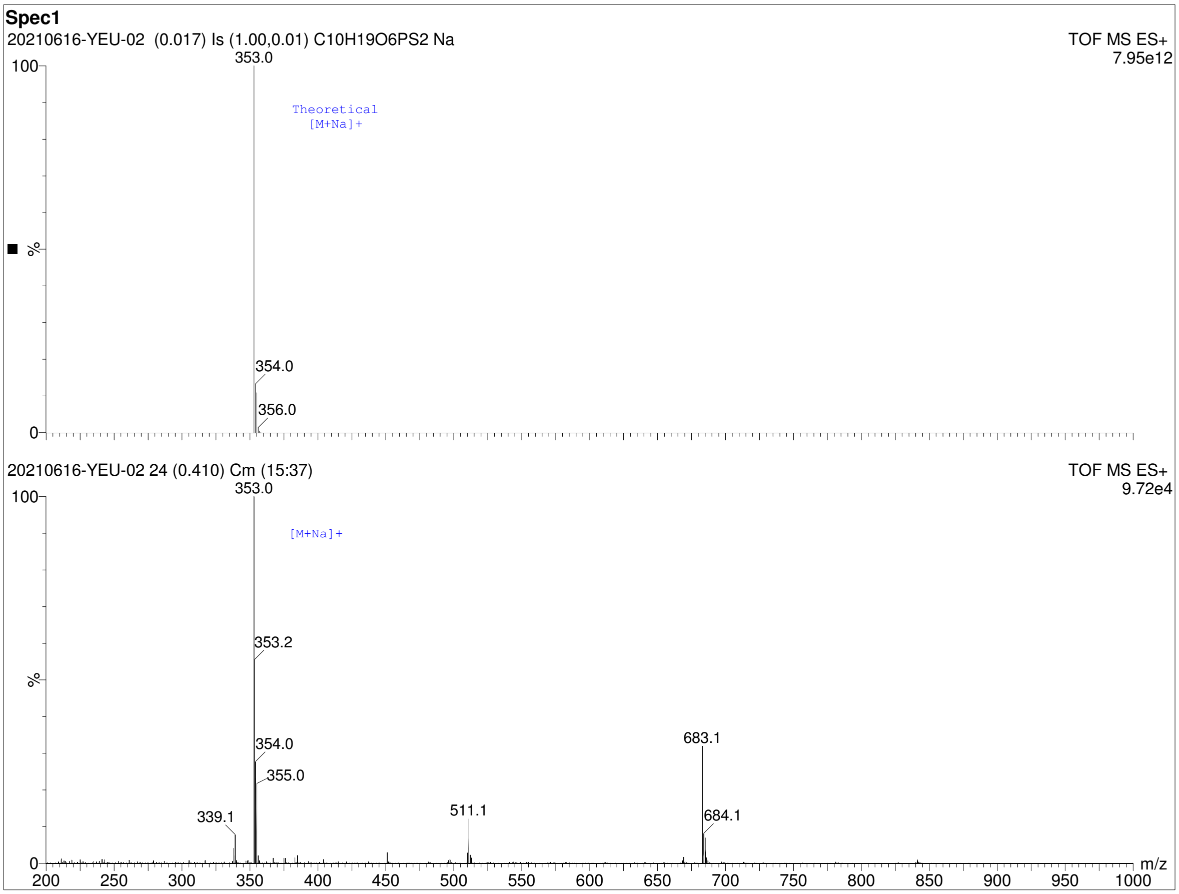

Figure 15: Mass spectrum of Spectracide (replicate 1) (Spectracide Catalog no. 071121309006) given by time-of-flight mass spectrometry. The theoretical mass spectrum of malathion is shown in the upper spectrum and the measured mass spectrum of Spectracide is shown in the lower spectrum.

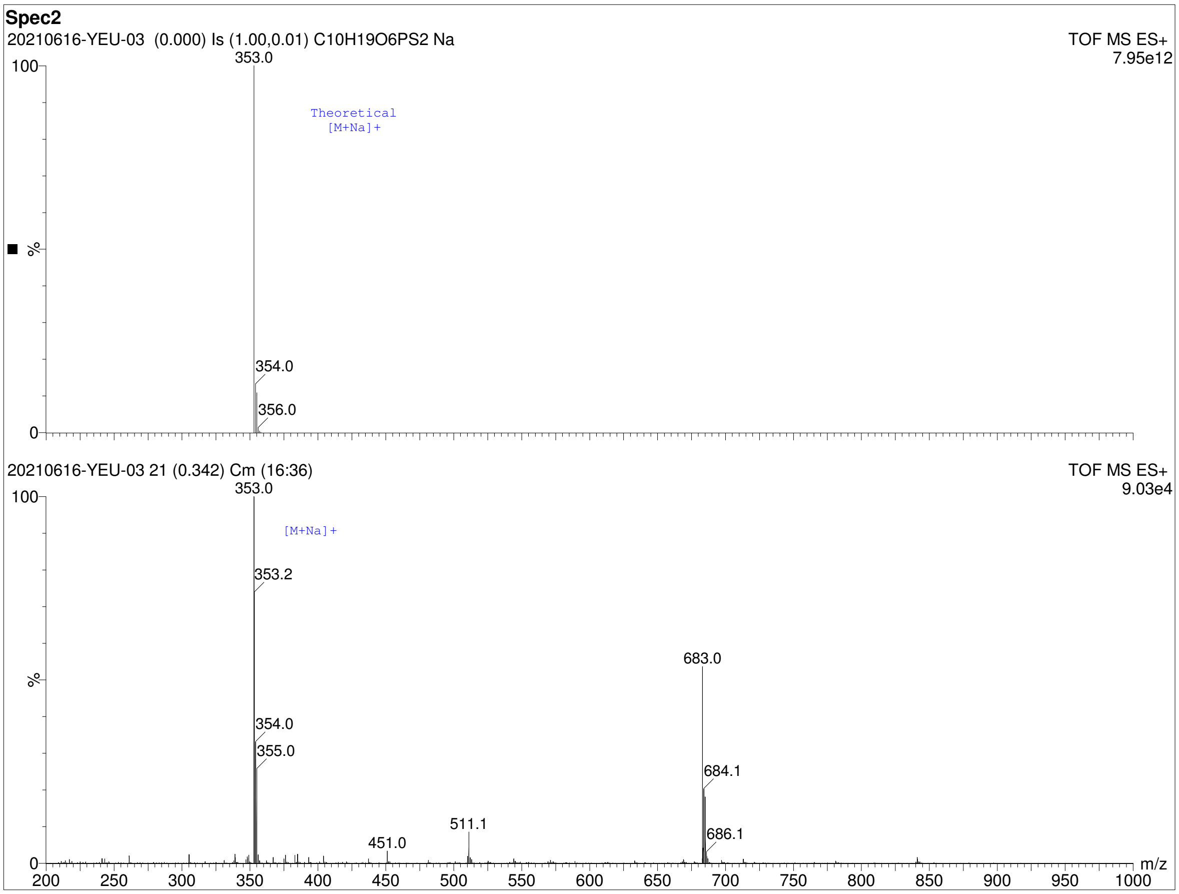

Figure 16: Mass spectrum of Spectracide (replicate 2) (Spectracide Catalog no. 071121309006) given by time-of-flight mass spectrometry. The theoretical mass spectrum of malathion is shown in the upper spectrum and the measured mass spectrum of Spectracide is shown in the lower spectrum.

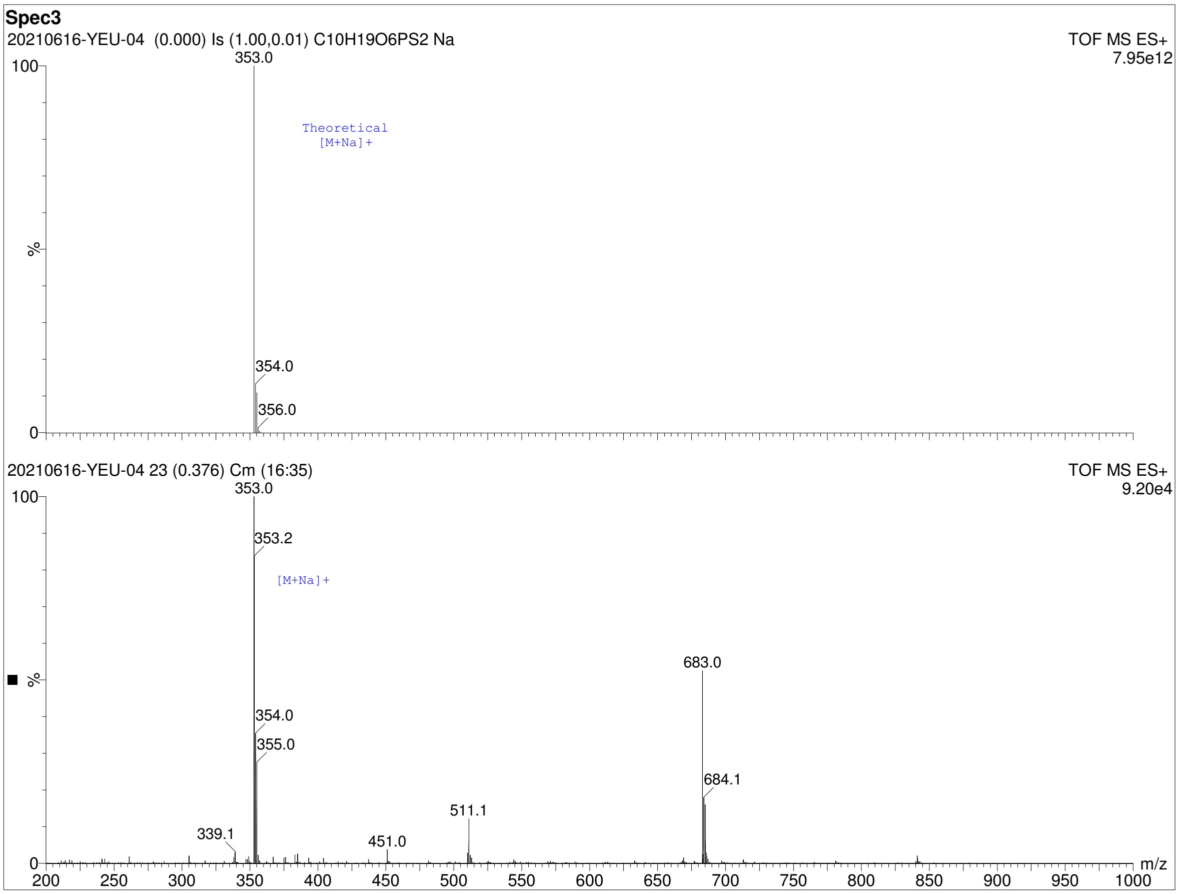

Figure 17: Mass spectrum of Spectracide (replicate 3) (Spectracide Catalog no. 071121309006) given by time-of-flight mass spectrometry. The theoretical mass spectrum of malathion is shown in the upper spectrum and the measured mass spectrum of Spectracide is shown in the lower spectrum.

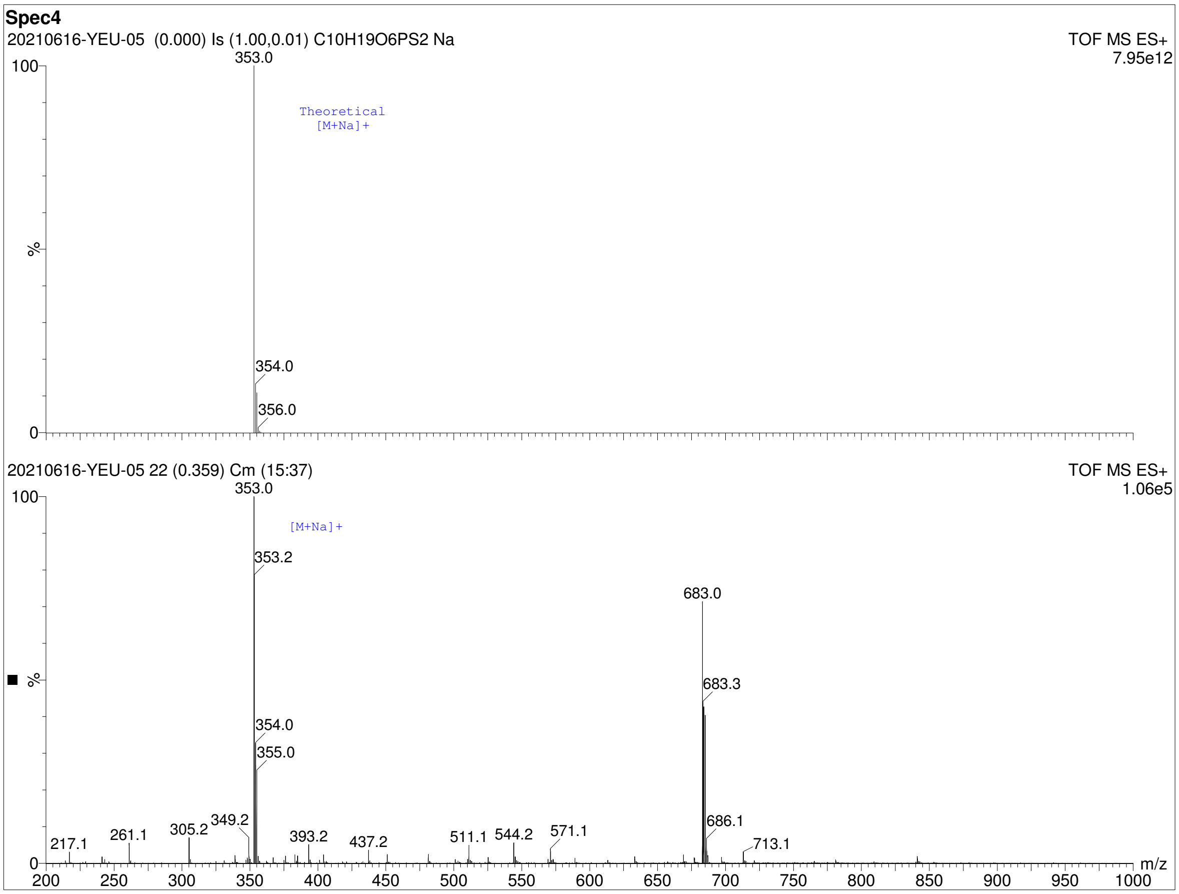

Figure 18: Mass spectrum of Spectracide (replicate 4) (Spectracide Catalog no. 071121309006) given by time-of-flight mass spectrometry. The theoretical mass spectrum of malathion is shown in the upper spectrum and the measured mass spectrum of Spectracide is shown in the lower spectrum.

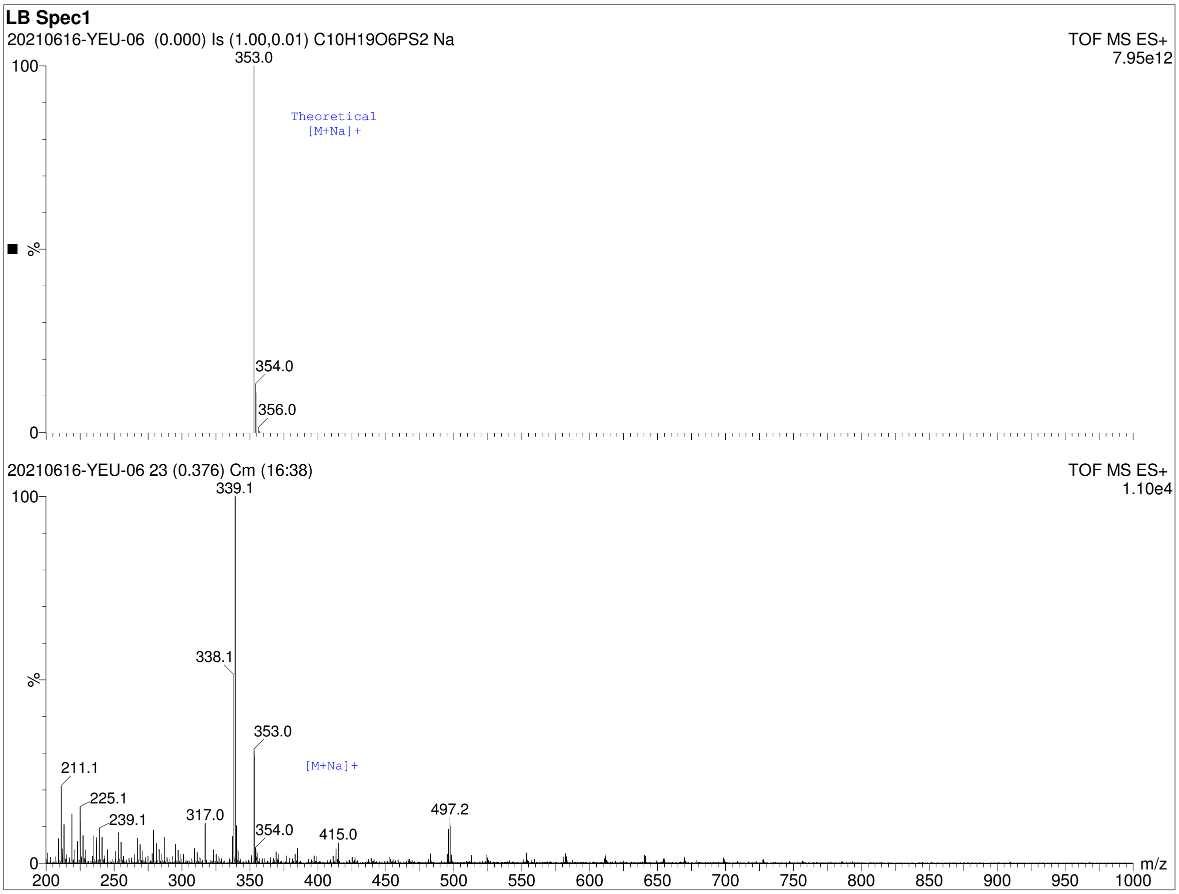

Figure 19: Mass spectrum of a 5% Spectracide in LB broth (replicate 1) given by time-of-flight mass spectrometry. The theoretical mass spectrum of malathion is shown in the upper spectrum and the measured mass spectrum of the solution is shown in the lower spectrum.

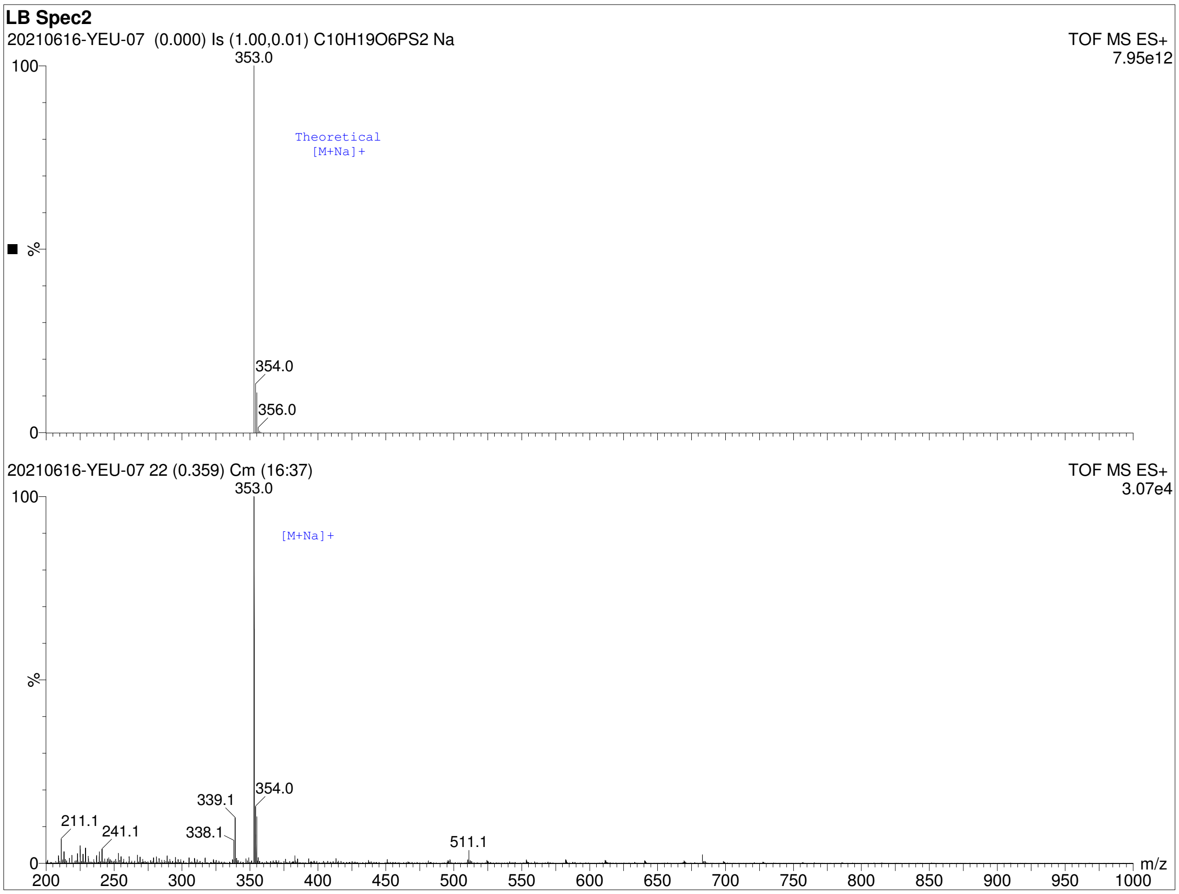

Figure 20: Mass spectrum of a 5% Spectracide in LB broth (replicate 2) given by time-of-flight mass spectrometry. The theoretical mass spectrum of malathion is shown in the upper spectrum and the measured mass spectrum of the solution is shown in the lower spectrum.

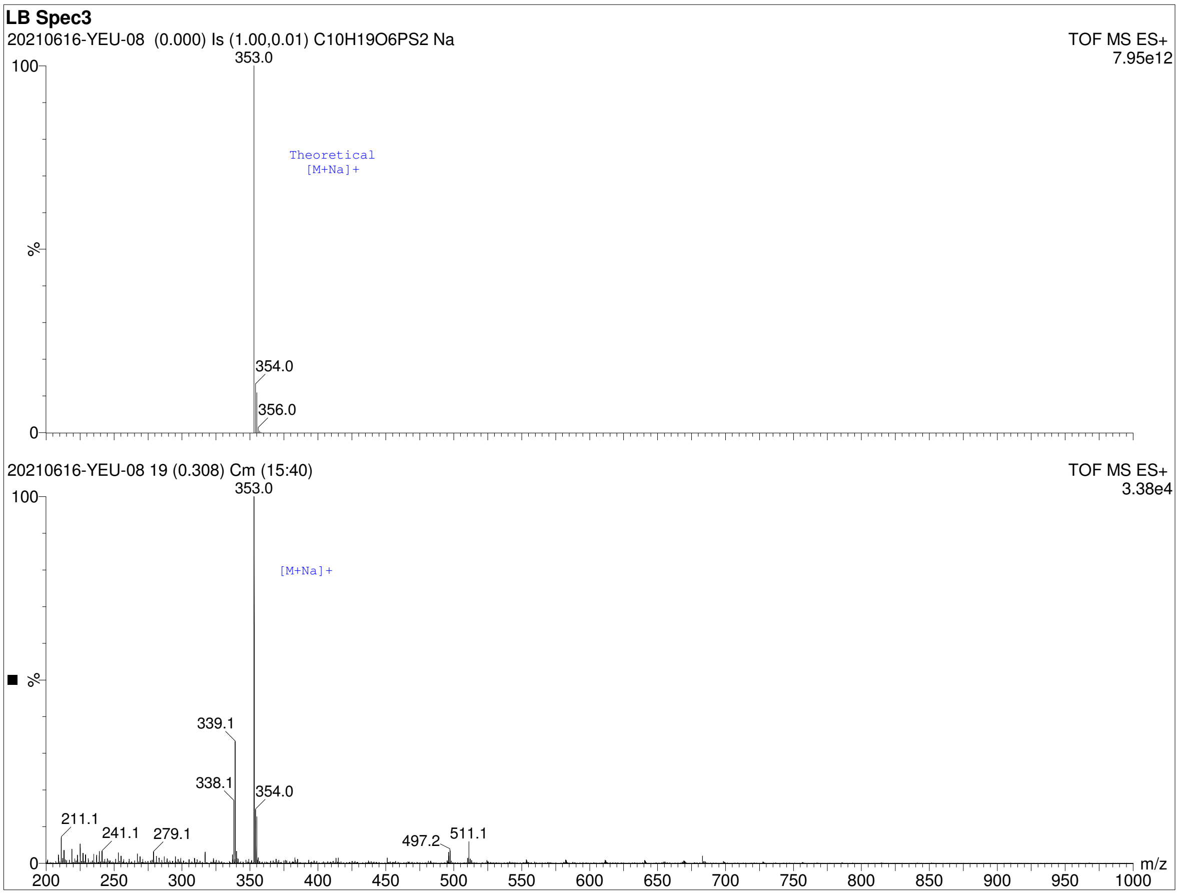

Figure 21: Mass spectrum of a 5% Spectracide in LB broth (replicate 3) given by time-of-flight mass spectrometry. The theoretical mass spectrum of malathion is shown in the upper spectrum and the measured mass spectrum of the solution is shown in the lower spectrum.

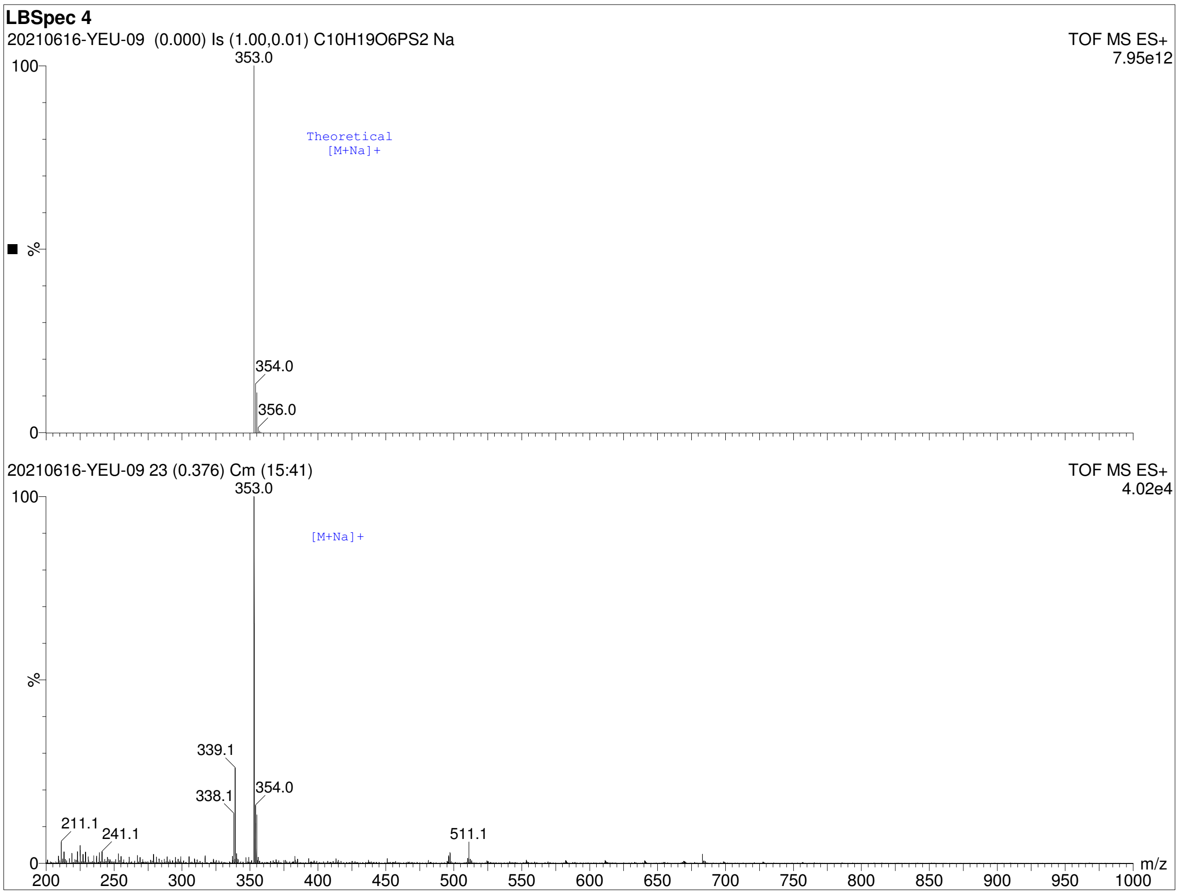

Figure 22: Mass spectrum of a 5% Spectracide in LB broth (replicate 4) given by time-of-flight mass spectrometry. The theoretical mass spectrum of malathion is shown in the upper spectrum and the measured mass spectrum of the solution is shown in the lower spectrum.

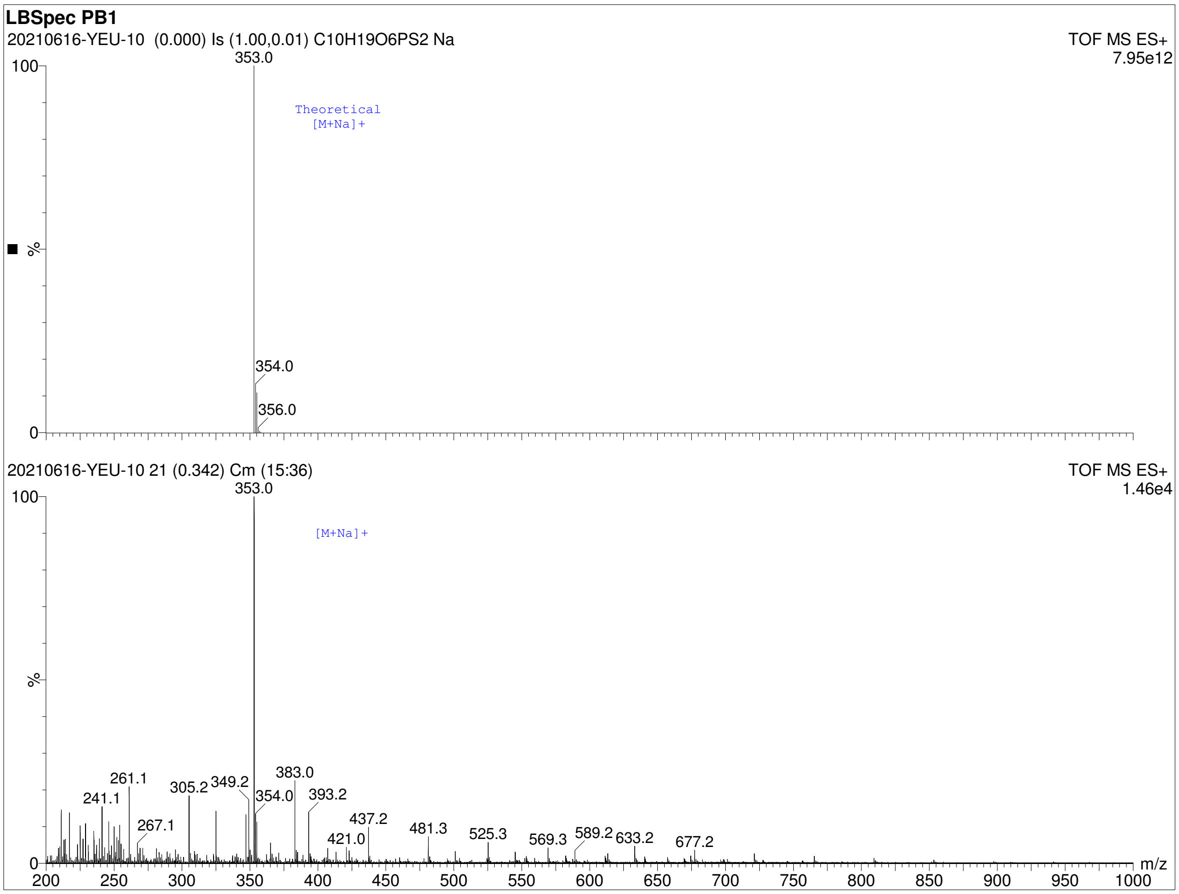

Figure 23: Mass spectrum of a 5% Spectracide in LB broth after photobleaching (replicate 1) given by time-of-flight mass spectrometry. The theoretical mass spectrum of malathion is shown in the upper spectrum and the measured mass spectrum of the photobleached solution is shown in the lower spectrum.

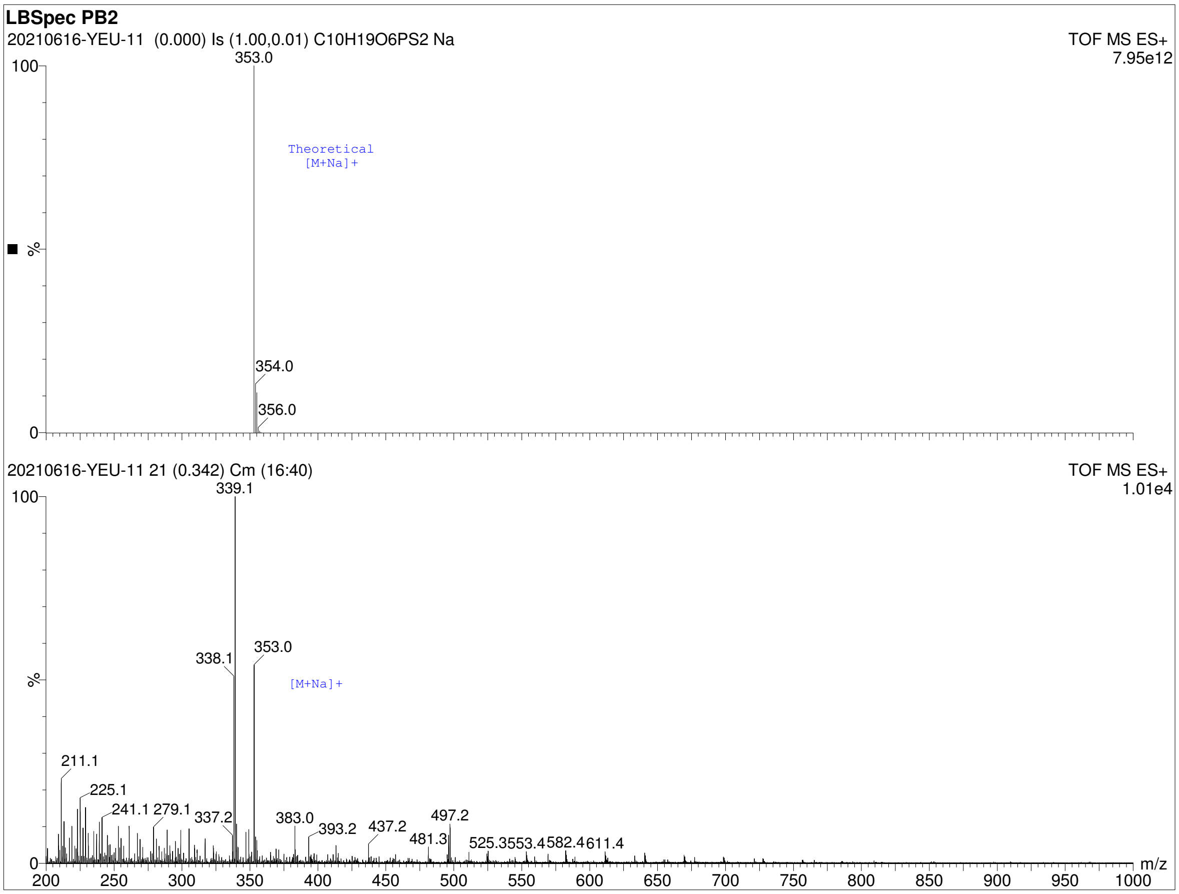

Figure 24: Mass spectrum of a 5% Spectracide in LB broth after photobleaching (replicate 2) given by time-of-flight mass spectrometry. The theoretical mass spectrum of malathion is shown in the upper spectrum and the measured mass spectrum of the photobleached solution is shown in the lower spectrum.

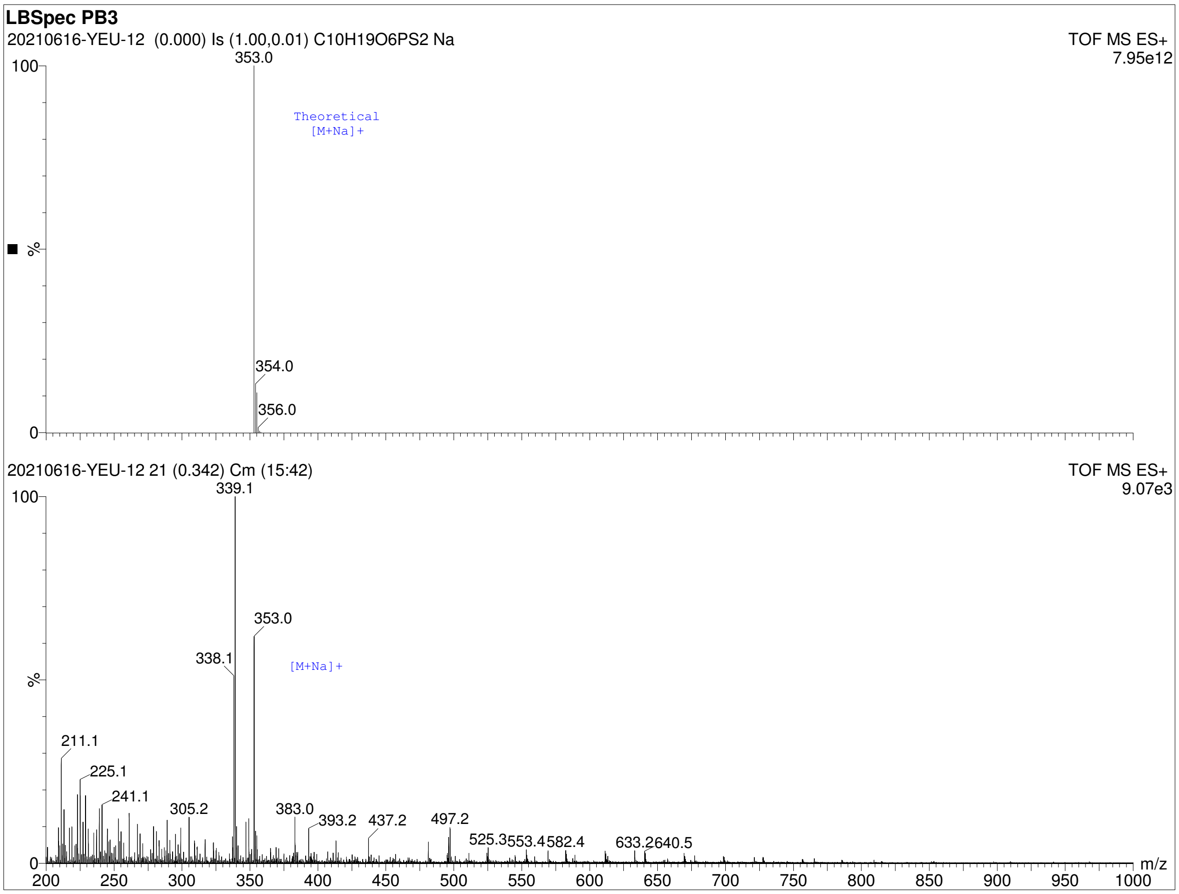

Figure 25: Mass spectrum of a 5% Spectracide in LB broth after photobleaching (replicate 3) given by time-of-flight mass spectrometry. The theoretical mass spectrum of malathion is shown in the upper spectrum and the measured mass spectrum of the photobleached solution is shown in the lower spectrum.

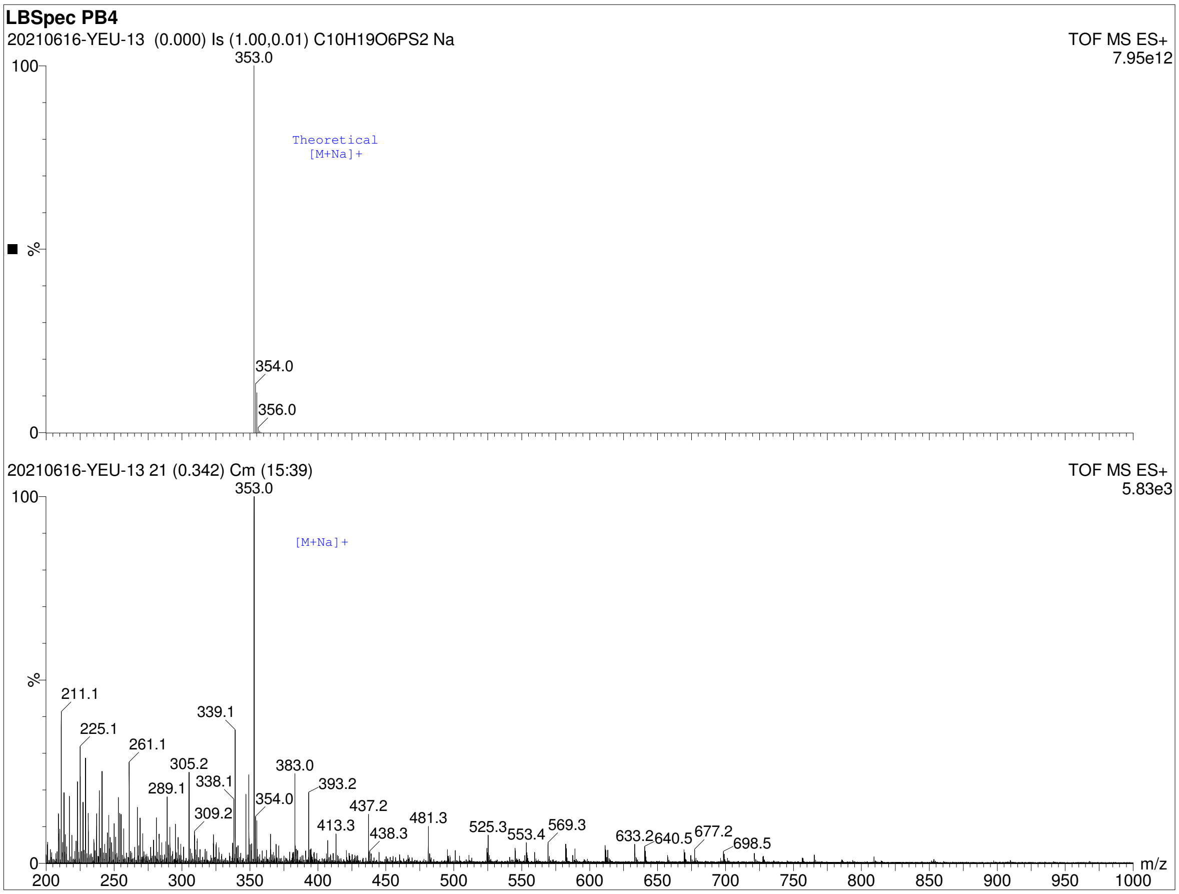

Figure 26: Mass spectrum of a 5% Spectracide in LB broth after photobleaching (replicate 4) given by time-of-flight mass spectrometry. The theoretical mass spectrum of malathion is shown in the upper spectrum and the measured mass spectrum of the photobleached solution is shown in the lower spectrum.

3 Supplementary Tables

Time point (minutes)	OD_{600}	Volume harvested (mL)	Malathion induction
0	0.5	10	
10	–	10	X
20	–	10	
30	–	10	
40	–	10	
50	–	10	
60	1.0	10	
70	–	10	
80	–	10	
90	–	10	

Table 1: Metadata for the time-series RNAseq experiment.

Sensor	Strand	Loci	Promoter sequence
PFLU_6124	Antisense	6709164 - 6709017	AACATTTGCTTATGTAGCGCGTGATCGGAAATCACTAC<br>CCGGCAGTTGAATAGGGGCAGAACCGCCCCTATACTCT<br>GCGCGCATTTTGTCGGCACAAATTATGCCAAGTTATTG<br>ATTTCCGGCAGCCGACCATTGAGGAGCAAGAGTG
PFLU_0841	Sense	950865 - 951131	TTCGCTTTACGTTCCACAAAAACGCCCAGCCTCCTCAC<br>GGAGCTGGGCGTTTTTATTGCCTGCGATTTATACACA<br>AATTTCGCCGTGACAACTTGCCACATCCGTAGACCCCC<br>TATACTACAAGGCCTGGAGGCTGAGCCCAGGGCAATTC<br>CCTTGTCATACGTGGGGCTTTTCATTACCATTCGGCAA<br>AATTTTTATAAGTAAAGATTCAACACTTAGTAGACGCC<br>TGATTTAACAGGCCAAAAAAGCTGATGGGAGAGGACT<br>GA
PFLU_4736	Sense	5213048 - 5213188	AGTGCTGGCAGAGGACGCTGGGTTTTTCTACACTGTGC<br>ACGAGATATTCCGTGCGCAGATTTATTGTCATTCGCGC<br>CTAAAGTTCGTCCGGGTATTGCCGAAAACATGGCAAGC<br>GTCCAAATACCCAGAGGTTTTTTGATC
PFLU_1823	Sense	1989934 - 1990137	GCGAGATAATAAGAAACCACGGCGGAGTTGCCCGTCG<br>TGAGCCTTGCGCGCAAGACTCACCGCGGAATATCCGCT<br>GGACGCAGTCTTGCGCAGCTTTACGGGCCTTGAGCCCC<br>GCAAGCTGCGCAAGCAGCAGTCACAGGTGGCGCGGCA<br>CTCATAATGAGCGCAGCGCCGAATGCGCAGTACCTAAC<br>GAAGACGGTAAAAAGC
PFLU_3761	Antisense	4158693 - 4158135	CTGTGACACGTCGCCAAGGCAGGCGCGGCGGATAGTT<br>TCAGTTCGGCGTCATACAAGTGCACTGCACCCCACTTC<br>ATCGTGGCCGTTTGCGAAAGCGATTGTCCGCTTGCGAC<br>GCGGCACAATCAGGGTATGTGCGCAGCTTGGCTTCCCA<br>GTAATTGCCCCATTAAATTTGTGGCTTTTCTGACGAGC<br>TTTTACTCGTCATCTCTTTGTTTTTTTACTATTATCGT<br>TCACCTGCGCACTCAAGGAAGAGAGGCTGAGCGCCTTG<br>AGGCTGGTAGAAAATTCATACTCGATCACTGAACGAGT<br>TATTGCTTTTACCCAGAACCTAACGACTCAGCCAACCA<br>TAAATACCTCTTGGTGAAACCGATGGATAAAATGTGTG<br>GCATCGTTGTAGTGGTAGGAC
PFLU_5502	Antisense	6038217-6038089	GTGTGATCCGCTTGAAGCCCGGCAGCTAGTGCGCTGCC<br>GGGTTGATTATTTGTTATTACAGCGATATTATCTCGCG<br>CCCTATTTCTTGGCTTCCGGGGCGTAGGTAGCTGTCAA<br>TTGGAGTCCCACTGA

PFLU_1836	Antisense	2003829 - 2003581	CGCCGCGCCATCAGCCAACTCCGACTGGCGTGAAAGAC<br>GAAAGTGCGGCAGTCTTAGGCACCCGAACGGGCCCAT<br>AAACAGGCCCGGTTTAAAATTTCAGTGAACAAGTGTA<br>CATTCAGTACCTTGCCGCTGTGACTTTCACTACAACGC<br>AATAGTCTATGTGTAGGCTGCCGACATGAGGCATGAAC<br>GCTTCATTCGGTTCGGGAAGATTTGCCCTACCCTGCCG<br>CATGGGATTATTGAGGAGCTCGC
PFLU_0376	Sense	417961 - 418174	AAGTCATAACTGCTTACACATCAACCGGTTGCCGGTAC<br>TCCTCTGCGTAAGTGTCTGCCCCTGAGCTTTGCCGCAC<br>CGATGTGGGGCTTTTCCGACATATGCCGATAACAAATA<br>GCCGTGAAACCTTTGTGACGAGCAACGAGTGGGTTAG<br>GATCGCACCCCGAAATGGTGCAGCCCTTTTGCGCGCCG<br>ACCTTACAAAAATCGTTCAGGGGAC
PFLU_1815	Antisense	1980804 - 1980440	GGCTTTTTTTCACACTGAAGAGCCCCTAACAATCAGGG<br>CAAAGTTGTTGGGGAGTGCGACTGGTCAGGTAAGCAC<br>CACCCAGGGAGTGCGACCCCCAATGAAAGCAAGCCCA<br>AAAGCCCTTGGCGGTCGGTGGCCGAGTATAGACAGTT<br>AGGTTACTAATGACAACGCGCACTCCTCACCCTAATAG<br>CTGATTGCGCTGGCGGGATAAAAGGCGTAAATGGCGC<br>TCAATTTCGAGGAAAAAGTACGGTTAAAGCCTTCTGGG<br>GCAAGACTTTAGGCAAATTGACATCTGAATTTATCTCA<br>CTATAGTGGTGCGGGCCCTGCGTGGGGGGTCTGTCTG<br>ATGATTTGAAGCATAAATAGGAGGCCAC
PFLU_0953	Sense	1058342 - 1058453	TGGAATGTATCAGGGCTATGAAGGTGATTGGTGTTCA<br>GCAAAGGTCTGGTCTGCTATTATCGCCAGCCTTTGTTG<br>ATACCAGTTCGCAATTTGCGCTGAAGCGGTCCAAGCC
PFLU_1380	Antisense	1527252 - 1526967	GGCAGTAAAACCTCAATCAGGACACTGGGGGCTATCG<br>TTAACGCAACGTTAATAGACGTAAACGATCATCCGAAT<br>ATTTGTGGGACGACACCGTCATGGGTGCCGAACGTAAT<br>GGAATCGAGGCTTCGGGCGTTGCTTTGTCAACACTCCG<br>CGAAGCCTGTCAAGAGTTTACAAACAACCATGAACGTA<br>AGTATATTGCGTAGCAAGCTACTTATCCACTCACAGCT<br>TGTTTTTTACCCTTCCACACTTCTTGTGCGCACCCTGC<br>GCGCCTGACCCGAGGATCTTC
PFLU_4612	Antisense	5088655 - 5088549	TGCGTGGCAAATATCTCTTACGTGTAGGCAAGTTCTGT<br>TAGACTTGTCGCCGAGTTGTCCCCCGGTTTGTGGGACT<br>GCTTTACAATCACCAGATGGGGATTTAACGG
PFLU_4150	Sense	4592631 - 4592843	GATTTGCCGCTGATCTCACGGCTTTTTTGGCGGTAAAA<br>CAGGCTTAAAACTGCCGCTTCTCACAAATTACGCAGCT<br>TTTACGGCTTTTTTTACCAGTTGATATTTCGAGCCAAA<br>GCCCCGTAAATCGGGGCTTTCAGCCGGATCAGGCACTA<br>AACGCAACGCTATTGATTAGCAAACAATGCCTTGGGGG<br>GGCTCCCAAGCCGAACATTTCGACTATGATAGCCCGGT<br>GTGCCCAGTTGGCCTGAGCAGCACAGCACTACTGAAAA<br>TATATGTTTCTTGGAGATACACC
PFLU_1302A	Antisense	1440968 - 1440759	GGCGGGTGTCCTGAATAGCGAGGTGGAAAAATACTGT<br>GGGGCATCTTACCGGGGCCGGCGTTGGGGTTCAAAGG<br>TCACAGGGCTTTTCTTGATGAATGCGCCGGCGGCTATA<br>AGCCGCAGCCAGCGGGGCCGGGGTTAATATTGCCGCG<br>CAGGGCGACCGTGGATACCACCGTCAGTCACGAATTTA<br>GAGAAACCTTCGGAAATACCATTGGCACGTTCCGGAAA<br>AAGGGTTAAGGTGGCGCCACTGTGCTGCTTGTGTCACT<br>GAGAATCTCTACACGATATGTTGAATTTCGATCCAACC<br>ATCTCCAAGAATTTTTCCTGCTCTTTGCACTCAGTCTC<br>GGCCAGGGCTTTTCCTGAGTCGCAGTTAACTTTGTCCA<br>AGGAGATACACC

PFLU_1358	Sense	1498195 - 1498311	AACAGCCTGCATCCATTGATGCAGGTCAGTTATTGCCC TTCTTTACGCTCCGTCGTGGGCGACATTGATCCCCGTC AATTTTCCAATCCGCCTTCTGCATTAACTTAGCCCTAT CGCAACAGGGCAAGTGCAGGAGGCCGGTC

Part	Sequence
BCD1	GGGCCCAAGTTCACTTAAAAAGGAGATCAACAATGAAAGCAATTTTCGTACTGAAACATCT TAATCATGCACAGGAGACTTTCT
ECK120033736	AACGCATGAGAAAGCCCCCGGAAGATCACCTTCCGGGGGCTTTTTTATTGCGC
sfGFP	ATGCGTAAAGGCGAAGAGCTGTTCACTGGTGTCGTCCCTATTCTGGTGGAACTGGATGGT GATGTCAACGGTCATAAGTTTTCCGTGCGTGGCGAGGGTGAAGGTGACGCAACTAATGGT AAACTGACGCTGAAGTTCATCTGTACTACTGGTAAACTGCCGGTACCTTGGCCGACTCTGG TAACGACGCTGACTTATGGTGTTCAGTGCTTTGCTCGTTATCCGGACCATATGAAGCAGCA TGACTTCTTCAAGTCCGCCATGCCGGAAGGCTATGTGCAGGAACGCACGATTTCCTTTAAG GATGACGGCACGTACAAAACGCGTGCGGAAGTGAAATTTGAAGGCGATACCCTGGTAAAC CGCATTGAGCTGAAAGGCATTGACTTTAAAGAAGACGGCAATATCCTGGGCCATAAGCTG GAATACAATTTTAACAGCCACAATGTTTACATCACCGCCGATAAACAAAAAAATGGCATTA AAGCGAATTTTAAAATTCGCCACAACGTGGAGGATGGCAGCGTGCAGCTGGCTGATCACT ACCAGCAAAACACTCCAATCGGTGATGGTCCTGTTCTGCTGCCAGACAATCACTATCTGAG CACGCAAAGCGTTCTGTCTAAAGATCCGAACGAGAAACGCGATCATATGGTTCTGCTGGA GTTCGTAACCGCAGCGGGCATCACGCATGGTATGGATGAACTGTACAAATGATGA
5' overhang	GAACGGTCTCAGCAT
3' overhang	GTCGTGAGACCTTACG

Table 3: Sequences for the parts used in the reporter cassette.

Malathion reporter	Locus tag	Time point (hours)
atpB	PFLU_6124	1.0
petA	PFLU_0841	2.0
anti-sigma 28 factor	PFLU_4736	3.2
sucC	PFLU_1823	8.1
Uncharacterized protein I	PFLU_3761	12.9
rpoA	PFLU_5502	15.0
fabA	PFLU_1836	14.0
Putative ABC transport protein	PFLU_0376	0.9
gltA	PFLU_1815	3.2
lpxC	PFLU_0953	0.7
acrA	PFLU_1380	3.1
Putative outer membrane porin A protein	PFLU_4612	2.0
cspA2	PFLU_4150	2.4
capB	PFLU_1302A	8.5
Uncharacterized protein II	PFLU_1358	5.6

Table 4: The time points at which the Hill functions are fit to each reporters' response. For each strain, the time point was chosen to be when the maximal fold change is achieved, where the fold change is of the 2.24 μM malathion response with respect to the 0 μM malathion response.
